# Merging konjac glucomannan with other copolymeric hydrogels as a cutting-edge liquid raft system for dual delivery of etoricoxib and famotidine

**DOI:** 10.1080/10717544.2023.2189630

**Published:** 2023-03-17

**Authors:** Nabil A. Shoman, Marwa Saady, Mahmoud Teaima, Rehab Abdelmonem, Mohamed A. El-Nabarawi, Sammar Fathy Elhabal

**Affiliations:** a Department of Pharmaceutics and Industrial Pharmacy, Faculty of Pharmacy, Ahram Canadian University, Giza, Egypt; b Department of Pharmaceutics and Industrial Pharmacy, Faculty of Pharmacy, Cairo University, Cairo, Egypt; c Department of Industrial Pharmacy, College of Pharmaceutical Sciences and Drug Manufacturing, Misr University for Science and Technology (MUST), Giza, Egypt; d Department of Pharmaceutics and Industrial Pharmacy, Faculty of Pharmacy, Modern University for Technology and Information (MTI), Mokattam, Cairo, Egypt

**Keywords:** Etoricoxib, famotidine, dual delivery, raft forming system, glucomannan, X-ray imaging

## Abstract

This study aimed to formulate and evaluate a floating raft system for the co-delivery of etoricoxib (ETO) and famotidine (FAM) using a combination of glucomannan with natural/semi-synthetic polysaccharides. Formulation variables affect gelation lag time (GLT), floating lag time (FLT), and release percentage of drugs after 1–8 h, Stability, and viscosity parameters were evaluated. In vivo X-ray studies, followed by the pharmacokinetic study, were performed on human volunteers. Formulations exhibited pseudoplastic behavior for ease of swallowing. The optimum raft system (ORS) comprised 1% Na alginate, 0.1% Low Methoxyl (LM) pectin, 0.8% Konjac glucomannan (KGL), 1% Precirol, and 1% CaCO_3_. ORS exhibited rapid GLT and FLT (around 42 and 8 sec respectively) in 0.1 N HCl as well as controlled release of ETO (15% in 1 h and 82% in 8 h) and FAM (29% in 1 h and 85% in 8 h). Formulation stability with the absence of any drug-excipient interactions was observed. The X-ray imaging showed a promising buoyancy ability for approximately 8 h. Compared with marketed products, ORS showed superior relative bioavailability for both drugs. These findings revealed the successful preparation of a promising raft system with improved dual drug delivery.

## 1. Introduction

Etoricoxib (ETO) is considered a selective cyclo-oxygenase-2 inhibitor, a sub-class of non-steroidal anti-inflammatory drugs, that obstructs prostaglandin production by inhibiting cyclo-oxygenase enzymes, thus reducing pain, discomfort, and inflammation resulting in an analgesia effect (Ahmadi et al., 2022; Farag & Bahra, 2022; Ju et al., 2022). Multiple clinical trials have demonstrated the clinical efficacy of ETO in treating acute and chronic pain associated with different diseases like acute gout, dysmenorrhea, oral surgery, rheumatoid arthritis, osteoarthritis, and chronic lower back pain (la Torre et al., 2021). The assessment of its side effects showed some cardiovascular risks, different renal events, dizziness, headache, and GIT disturbances like nausea, vomiting, and peptic ulcer (Kirschneck et al., 2019; la Torre et al., 2021). Although it exhibits some GIT problems and peptic ulcers, evidence-based studies showed that these effects are well tolerated and less likely to happen in contrast to ibuprofen or nonselective NSAIDs (Martina et al., 2005; Yuan & Hunt, 2007).

The efficacy and safety profile of ETO plus the frequent use of analgesics that may cause severe GIT disturbances and ulcers have contributed to the evolution of this research work. Methods to reduce the drug’s GIT side effects like the incorporation of antacids (H_2_ blockers) (Birk & Myers, 2009), and designing the floating raft systems (RS) (in-situ gelling approach) were proposed. Clinical evidence has proven that famotidine in combination with NSAIDs can be used to minimize the GIT side effects resulting from the frequent use of analgesics (Birk & Myers, 2009; Bello, 2012; Deeks, 2013; Sugano, 2013; Bello et al., 2015; Taha, 2015). Famotidine is a selective H2 receptor antagonist that is used to treat GIT ulcers by inhibiting both the concentration and volume of gastric acid secretions (Bello, 2012).

The RS is a promising delivery system among the other liquid oral controlled release systems as the floating capability of RS is higher than the conventional floating formulations. At room temperature, it is a fluid-like that incorporates an in-situ gel-forming biopolymer (sodium alginate or LM-pectin) with an agent that forms gas (carbonates or bicarbonates). Upon any change in pH or contact with the gastric fluids, the former liquid mixture swells, creating a viscous adhesive gel layer that could trap CO_2_, lowering the density and thus enhancing the buoyancy of the RS on the surface of gastric fluids without affecting gastric emptying (M. H. Teaima et al., 2020). Thus, the liquid raft system is more patient-tolerable and easy to swallow, targeting more geriatrics and pediatrics.

In this RS study, lipid excipients and potential combinations of glucomannan, other natural gums, semi-synthetic hydrogels, and lipid excipients (Precirol® ATO5 (Pr)) have been integrated. Lipid excipients (such as Precirol^®^ ATO5 (Pr)) have better characterization, inertness properties, versatility, and enhanced sustained drug releases (Rosiaux et al., 2014). Natural biopolymers and polysaccharides possess versatile and biodegradable characteristics, rendering them to be commonly applicable and significant in extended-release delivery systems (Prajapati et al., 2013; Szekalska et al., 2016; Xu et al., 2021).

As recent studies suggest the favorable effect of alginates in reducing heartburn and some gastrointestinal acid refluxes, incorporating a specific number of alginates would have a synergistic effect with famotidine to treat and minimize any gastrointestinal adverse events. This possible effect is established by the blocking action of the alginate-based raft, as it acts as a barrier to acid and food reflux. This takes place through the alginates mechanism of displacing the postprandial gastric acid pocket (Yamamoto et al., 2014; Leiman et al., 2017; C.-X. Zhao et al., 2020).

Konjac glucomannan (KGL) is a natural, soluble, highly viscous polysaccharide fiber that is extracted from the tuber of Amorphophallus Konjac roots (Vaughn, 2012). KGL exhibits some medical characteristics like lowering cholesterol, treating constipation and diabetes, and promoting weight loss (Vaughn, 2012). Moreover, KGL can possess some anti-inflammatory effect that would synergize the effect of etoricoxib in managing pain and reduces the inflammatory mediators (Y. Zhao et al., 2020; Wei et al., 2022). One of the suggested mechanisms is the regulation of the nuclear factor kappa B pathway and possible reduction in the population of these inflammatory cells thus preventing oxidative stress and reducing inflammation (Devaraj et al., 2019; Y. Zhao et al., 2020). The other mechanism is the down-regulation of the inflammatory factor, tumor necrosis factor α, which is a potent mediator of inflammatory and immune functions (Behera & Ray, 2016; Wei et al., 2022).

On the pharmaceutical industry side, KGL is considered a novel excipient that can be used as a tablet disintegrant, filler binder, and film-forming agent, and encapsulates compounds or drug carriers for controlled release. Besides, it possesses some food industrial benefits as a gelling component in jelly-food products. This is due to its strong hydrophilicity and extremely high viscosity features (Aanisah et al., 2022).

The statistical experimental designs have been proposed as an efficacious way to formulate and optimize new drug delivery systems with few trials and little cost. Thus, these designs of experiments with applying the desirability functions have been utilized to suggest the composition of the best-achieved formulations (Lewis et al., 1998). In this work, the 2^4^ full factorial design was used for inspecting the influence of the formulation variables.

This study aims to implement the promising KGL polymer in the design, formulation, and optimization of the ETO/FAM combined RS to be one of the superior research projects in targeting gastric ulcers associated with the excessive use of analgesics. The best-chosen system would provide extended drug release, the lowest possible time for intact gelation and buoyancy with comparable bioavailability to the marketed tablets. The incorporation of sucrose in this formulation makes it palatable and thereby enhances patient compliance, thus rendering it the ideal candidate for all patients who have swallowing difficulties, especially pediatrics, and geriatrics.

## 2. Materials

Etoricoxib and Arcoxia^®^ 60 mg tablets (Reference; Batch no 31099) (Merck, Sharp & Dohme, Cairo, Egypt), Famotidine and Antodine^®^ 20 mg tablets (Reference; Batch no 3728) Amoun, Cairo, Egypt, Konjac Glucomannan Powder (Hubei Yizhi Konjac Biotechnology Co., Ltd, Yichang, China), Low Methoxyl (LM) Pectin (Xian Ceres Biotech Co., Ltd, Hunan, China), Sodium (Na) alginate (General chemical & pharmaceutical Co. Ltd, Sudbury, England), Glyceryl palmitostearate (Precirol^®^ ATO 5) (Gattefossé S.A., Lyon, France), Sucralose, Ca Carbonate (CaCO_3_) and Sodium (Na) Citrate, (EIPICO Company, Cairo, Egypt), another chemical ingredients and solvents were of the analytical grades.

## 3. Methods

### 3.1. Preparation of ETO/FAM raft systems (RS)

The prepared RS were liquid solutions with appropriate in-situ gelling and floating behavior to attain controlled drug release formulation with better patient compliance. Disparate quantities of Pr were mixed with polymeric hydrogels to slow down the drug release rate. The water bath’s temperature was maintained at 90 °C in which Pr was melted and ETO/FAM was dispersed in the melted lipid (60 mg/ml & 20 mg/ml) for each formula. Briefly, Na alginate (1% w/v) and hydrogel enhancers (0.1% w/v) followed by KGL polymer were sprinkled and solubilized in deionized water. Then the fixed concentration of Na citrate (0.25% w/v) was dispersed to the solution. The solution’s temperature was raised to the same temperature of melting lipids along with continuous agitation till a congruent viscous liquid-gel was formed and then poured to ETO/FAM lipid dispersion. Additionally, a satisfactory concentration (0.5% w/v) of sucralose was included to cover up the pungent taste of the drugs. Finally, two distinct amounts of calcium carbonate were distributed to formulations and blended for 10 min using a homogenizer, at 2000 rpm till achieving a steady strong uniformed emulsion (Ibrahim, 2009).

### 3.2. Factorial design statistical arithmetic

Using the Design Expert^®^ program (Version 7, Stat-Ease Inc. Minneapolis, MN, USA), a 2^4^ full factorial design was integrated to examine the potential effect of the multiple variables on the conforming responses. The independent variables studied were as follows: X_1_: type of hydrogel enhancer, X_2_**:** KGL concentration, X_3_**:** glyceride lipid concentration, and X_4_: CaCO_3_ concentration, each at two levels as shown in Table 1. The preliminary trials were fundamental for the establishment of the lower and upper constraints of each variable’s concentration. Sixteen formulas and their components are presented in Table 2.

**Table 1.** 2^4^ full factorial design for the formulation of ETO/FAM floating raft systems (RS): factors and responses.

Factors (independent variables)	Levels		
	Low	High	
X1: Type of hydrogel enhancer	Gellan gum	Pectin	
X2: KGL concentration (%)	0.5	0.8	
X3: Conc. of glyceride lipid (%)	1	2	
X4: Conc. of CaCO_3_ (%)	1	2	
Responses (dependent variables)	Desirability constraints	Adjusted R^2^	Predicted R^2^
Y_1_: Gelation lag time (sec)	Minimize	0.945	0.812
Y_2_: Floating lag time (sec)	Minimize	0.953	0.838
Y_3_: ETO release (%) after 1 h	10 %≤ Y4 ≤ 20 %, in range	0.985	0.948
Y_4_: FAM release (%) after 1 h	20 %≤ Y4 ≤ 30 %, in range	0.935	0.777
Y_5_: ETO release (%) after 8 h	70 %≤ Y4 ≤ 90 %, in range	0.977	0.920
Y_6_: FAM release (%) after 8 h	80 %≤ Y4 ≤ 90 %, in range	0.930	0.762

**Table 2.** Design of the prepared ETO/FAM RS in actual values (uncoded units) and their calculated responses.

	Factors								Responses	
	X_1_	X_2_	X_3_	X_4_	Y_1_	Y_2_	Y_3_	Y_4_	Y_5_	Y_6_
**RS-1**	Pectin	0.5	1	2	90.56 ± 0.92	20.94 ± 1.57	23.32 ± 1.57	37.31 ± 0.071	85.98 ± 1.56	90.49 ± 2.07
**RS-2**	Pectin	0.8	1	2	56.25 ± 0.45	5.24 ± 0.55	12.77 ± 0.55	29.53 ± 0.12	56.57 ± 1.63	71.95 ± 1.06
**RS-3**	Pectin	0.8	2	2	27.06 ± 0.27	1.5 ± 0.28	8.83 ± 0.28	15.89 ± 0.53	41.55 ± 1.23	55.19 ± 2.55
**RS-4**	Pectin	0.8	1	1	42.29 ± 0.38	7.81 ± 0.67	14.94 ± 0.67	28.83 ± 0.21	82.04 ± 1.29	84.84 ± 1.11
**RS-5**	Pectin	0.5	1	1	89.56 ± 0.55	47.76 ± 1.14	31.45 ± 1.14	40.78 ± 0.11	95.72 ± 1.21	97.32 ± 2.05
**RS-6**	Pectin	0.8	2	1	69.05 ± 0.63	6.12 ± 1.61	8.78 ± 0.71	16.25 ± 0.06	50.33 ± 1.02	59.85 ± 1.16
**RS-7**	Pectin	0.5	2	1	81.75 ± 0.70	52.41 ± 0.18	24.57 ± 0.12	23.79 ± 1.02	78.46 ± 1.99	79.61 ± 1.83
**RS-8**	Pectin	0.5	2	2	50.12 ± 0.58	29.54 ± 0.14	22.76 ± 1.14	19.60 ± 1.12	63.9 ± 1.08	67.69 ± 1.89
**RS-9**	Gellan Gum	0.5	2	1	26.18 ± 0.18	34.98 ± 1.88	21.42 ± 1.08	26.18 ± 0.021	68.35 ± 1.22	71.63 ± 2.38
**RS-10**	Gellan Gum	0.5	2	2	24.94 ± 0.91	21.97 ± 1.13	7.57 ± 0.13	14.53 ± 0.019	55.4 ± 0.79	64.86 ± 3.01
**RS-11**	Gellan Gum	0.5	1	1	41.5 ± 0.21	14.87 ± 0.33	34.67 ± 1.07	44.14 ± 0.14	92.91 ± 0.85	96.12 ± 3.51
**RS-12**	Gellan Gum	0.8	1	1	33.82 ± 0.65	5.75 ± 0.46	28.95 ± 2.46	36.12 ± 0.031	84.04 ± 1.34	91.21 ± 3.17
**RS-13**	Gellan Gum	0.5	1	2	78.37 ± 0.33	11.5 ± 1.03	12.66 ± 0.49	27.06 ± 0.18	68.71 ± 1.15	77.98 ± 2.29
**RS-14**	Gellan Gum	0.8	1	2	59.31 ± 0.88	16.86 ± 1.06	14.81 ± 1.16	22.83 ± 1.28	68.39 ± 0.51	83.21 ± 3.16
**RS-15**	Gellan Gum	0.8	2	1	28.87 ± 0.66	7.94 ± 0.11	16.05 ± 0.01	20.8 ± 0.98	60.86 ± 1.09	65.56 ± 2.31
**RS-16**	Gellan Gum	0.8	2	2	30.85 ± 0.89	10.42 ± 1.53	6.515 ± 0.23	13.02 ± 0.18	47.08 ± 1.04	53.92 ± 1.77

Data are represented as mean ± standard deviation (*n* = 3).

Abbreviations: X_1_, type of hydrogel enhancer; X_2_, KGL concentration; X_3_: Conc. of glyceride lipid; X_4_, conc. of CaCO_3_; Y_1_, gelation lag time (sec); Y_2_, floating lag time (sec); Y_3_, percent of ETO released after 1 h (%); Y_4_, percent of FAM released after 1 h (%); Y_5_, percent of ETO released after 8 h (%); Y_6_, percent of FAM released after 8 h (%).

### 3.3. In vitro characterization of ETO/FAM RS

#### 3.3.1. Gelation study

The gelation capacity of the floating raft was assessed three times for each RS, by cautiously putting two milliliters in a graduated cylinder with 6 ml of the acidic buffer 0.1 N HCl (pH 1.2), as a gelation medium. All systems were freshly prepared and added slowly while equilibrated at 37 ± 0.5 °C [16]. The time required for the constitution of the gel (gelation lag time; GLT) was detected and the gel formulation capacity was perceivably assessed.

#### 3.3.2. Floating assessment

The capability of the prepared raft systems to float was assessed by examining both floating lag time (FLT) and floating duration (El Nabarawi et al., 2017). The assessment was performed in triplicate for every RS by placing a 10 ml raft system (placed into a watch glass) in a 250 ml beaker with 200 ml of 0.1 N HCl (pH 1.2) at 37 ± 0.5 °C. Their integral physical state and their buoyancy behavior were examined for 12 h.

#### 3.3.3. In vitro release studies

The release of ETO/FAM from the formulated RS was conducted by USP dissolution test apparatus II (USP 24). Nine hundred (900) ml of the acidic buffer 0.1 N HCl (pH 1.2) was added to the apparatus as the dissolution medium. At 50 rpm, the paddle stirrer was adjusted while the temperature was kept at 37 °C (M. Teaima et al., 2020). On a watch glass, ten milliliters of each RS were placed and then kept in the vessel with the lowest disturbance possible. At these fixed time intervals (0, 1, 2, 3, 4, 5, 6, 7, and 8 h), an exact computed sample of the dissolution medium was replaced with the exact volume of fresh medium kept at the same temperature. The absorbance of ETO and FAM in the taken samples was computed at 310 and 282 nm, respectively by using the UV spectrophotometer. These dissolution tests were held three times.

#### 3.3.4. Kinetic modeling on drug release

The drug release kinetics were investigated depending on zero-order, first-order, Higuchi, and Korsmeyer-Peppas kinetic paradigms (Dash et al., 2010). The paradigm which has the highest coefficient of determination (R^2^) is contemplated to be the best fitting for each system. This was performed with the help of an add-in program for Microsoft Excel (DDSolver) that was employed to model and emulate the drug delivery profiles (Y. Zhang et al., 2010).

#### 3.3.5. Viscosity measurement

A rotating viscometer was used to determine the viscosity of the prepared formulations using the appropriate spindles. At various angular velocities (10–100 rpm) and a temperature of 32 ± 0.5 °C, the RS viscosity was measured twice at 30 s at every two speeds (Rajinikanth et al., 2007). For the speculation of the rheological manner of each RS, the rheological data were tested by using Farrow’s equation (Farrow et al., 2004).

(1) Farrow′s equation: Log D = N log S-Log η.

where D: Shear rate (sec^−1^), S: Shear Stress (dyne/cm^2^), N: Farrow’s constant, and η: Viscosity (cP). N (Farrow’s constant) is the slope of log D against the log S plot, which indicates the deviation from the Newtonian flow. When N is less than one, dilatant flow is indicated, and if greater than 1, pseudoplastic flow is assured.

#### 3.3.6. Nomination of optimized raft system (ORS)

Using a 2^4^ factorial experiment design, six dependent variables were selected to study the effect of studied factors on each response. As shown in Table 1, the dependent variables were GLT (Y_1_), FLT (Y_2_), the percent of ETO released after 1 h (Y_3_) and 8 h (Y_5_), as well as the percent of FAM released after 1 h (Y_4_) and 8 h (Y_6_). The significance of each factor was evaluated using the Analysis of variance (ANOVA) model. The prediction of the optimized raft system was anticipated through the desirability approach regarding the minimum GLT and FLT with the controlled-release RS.

#### 3.3.7. Short-term stability study

The ORS was kept in sealed glass vials (30 ml) at 4 °C and 25 °C for 45 and 90 days to investigate the physical stability of the raft system. Samples from each formula were taken at 0, 45, and 90 days (Aashigari et al., 2019). At the end of the storage period, GLT, FLT, percentage of ETO released after 1 h, percentage of FAM released after 1 h, percentage of ETO released after 8 h, and percentage of FAM released after 8 h were determined as mentioned before and compared with the freshly prepared one, using a Student t test (at *p* ≤ 0.05).

#### 3.3.8. Differential scanning calorimetry

For the evaluation of any possible drug–excipient interaction, differential scanning calorimetry (DSC) analyses were performed (DSC60 TA-60 WS, Shimadzu, Japan) (A. R et al., 2021). The pure ETO, FAM, Na alginate, Pr, LM pectin, KGL, and physical form of ORS were the subject samples. An aluminum pan was loaded with 3–4 mg of the samples and then sealed. The analyzed powder sample was placed over the heating rate of 10 °C/min and temperature (25–300 °C) range. Then, a nitrogen gas purge was used to maintain the inert atmosphere in the system at the rate of 100 ml/min.

#### 3.3.9. Fourier transform infrared spectroscopy

Inside the formulations, there might be any potential drug-excipients-associated interactions. Thus, Fourier Transform Infrared Spectroscopy (FTIR) is commonly used to investigate any possible interactions (ELhabal et al., 2022). FTIR is generally employed to check any potential interactions between the drug and the excipients used in the formulation. The recorded spectrums were between 4,000 – 455 cm^−1^ for refined drugs, excipients, and the physical form of ORS. They were blended with 0.5 gm of IR-grade potassium bromide particles and pressed into a round disk intensively.

### 3.4. In vivo X-ray study in human volunteers

By the x-ray imaging technique, an in vivo radiograph examination was performed on healthy human volunteers, for the assessment of the gastroretentive behavior for ORS as per the protocol approved by the Institutional Ethical Committee, (PI 3014, dated June 28, 2021) at the Center of Applied Research and Advanced Studies (CARAS), Faculty of Pharmacy, Cairo University, Kasr Al-Aini Street, Cairo, Egypt. As per the declarations of Helsinki, the study was thoroughly justified to two female healthy volunteers before performing it, and the informed consent forms were signed by each volunteer (A, 2000; *WMA - The World Medical Association-WMA Declaration of Helsinki – Ethical Principles for Medical Research Involving Human Subjects*, n.d.). These two volunteers weighing approximately 70 kg were introduced to abdominal X-ray imaging. Before this, the volunteers ate a low-calorie meal after overnight fasting to avoid any troubles during the imaging. When the study was initiated, each subject was orally administered with the optimized formula of ORS containing X-ray grade barium sulfate (15% w/w) as a marker with 200 ml water (Moganti & Shivakumar, 2021). The volunteers were in the upright position for imaging to locate the position of the optimum formula in the GI tract. X-ray images were captured at zero time and then at pre-determined time intervals (0, 0.25, 2, 4, 6, 8 hours) post-administration. Volunteers had access to only water during the study’s conduction.

### 3.5. In vivo pharmacokinetics in human volunteers

#### 3.5.1. Experimental plan and samples assembling

An in vivo study was carried out to juxtapose the pharmacokinetic profiles of the ETO/FAM floating ORS (60/20 mg) and the commercially available products, Arcoxia^®^ 60 mg and Antodine^®^ 20 mg tablets (standard), after sole oral dose administration. Faculty of Pharmacy, Cairo University Research Committee approved the protocol and the informed consent form (PI 3014). The study involved six healthy volunteers (male Egyptian) of 18–50 years with heights of 160–175 cm with a BMI range from 18–35 kg/m^2^ were recruited for the study. The procedures were fully clarified to each volunteer at the start of the experiment and the informed consent forms were signed by each subject as per the declarations of Helsinki (Brazil 2013).

Two treatments, two periods, cross-over randomized design were performed along with a 1-week washout period to separate the two periods. Randomly, the volunteers were assigned to one of the two equally sized groups. The volunteers fasted for 10 h approximately and got water 1 h before the appointed time of dosing. The remedies were taken with 240 ml of water. With their acceptance, water was allowed after 2 h and a planned meal after 4 h from the two drugs administration.

Samples of venous blood (5 ml) were assembled into blood tubes containing heparin through the lodging cannula immediately before the oral administration and at the scheduled periods of 1, 2, 3, 4, 5, 6, 8, 24, 48, and 72 h after dosing. The withdrawn plasma samples were subjected to centrifugation at 3,500 rpm for 10 min and then were frozen at −20 °C in labeled tubes for further analysis.

#### 3.5.2. Sample arrangement

About 100 µL of celecoxib and 35 µL of pantoprazole (from a stock solution of concentration of 300 µg/ml, and 1 µg/ml) were added to each sample (0.5 ml plasma) as internal standards. ETO, FAM, celecoxib, and pantoprazole were extracted using ethyl acetate (3 ml), and vortexed for 1 min, then centrifuged for 10 min at 5000 rpm (cooling centrifuge, TGL-20 MB). The supernatants were transferred in other vials filtered through a 0.22–lm Millipore filter and evaporated to dryness using a vacuum concentrator (Eppendorf Vacufuge plus, Germany).

#### 3.5.3. LC-MS/MS assay of ETO/FAM

A validated LC-MS/MS technique for analysis of the drug concentrations in plasma was applied using LC-MS/MS system (Shimadzu^®^, Japan) coupled with a triple quadrupole detector (API-4500, AB Sciex, Foster, CA, USA). The mobile phase consists of 90% acetonitrile, 10% 0.01 M ammonium formate, and 0.1% formic acid in water. The chromatographic separation was executed on the Agilent poroshell 120EC-C18 2.7-Micron (4.6 × 50 mm i.d,5 µm diameter; CA, USA). The used injection volume was 10 µL and the isocratic flow rate was 0.3 ml/min.

#### 3.5.4. Statistical analysis of pharmacokinetic profiles

Following the intake of the oral ORS dose and the marketed product, a non-compartmental pharmacokinetic model was utilized via the PK solver program for the evaluation of the ETO/FAM’s pharmacokinetic parameters (Y. Zhang et al., 2010).

The assessed parameters were peak concentration (C_max_, ng/ml), time to reach the highest concentration (T_max_, h), elimination rate constant (K_el_, h ^− 1^), the area under the plasma concentration-time curve from time zero to the last observation time point (AUC_(0–72)_, ng. h/ml) and infinity (AUC_(0–∞)_, ng. h/ml). IBM SPSS Statistics 20 (Armonk, NY, USA) was used for analyzing the statistical differences in data through a one-way ANOVA test for the studied pharmacokinetic parameters, and the P-value <0.05 was statistically significant. Nonparametric Kruskal–Wallis test was performed to compare the T_max_ data obtained from the treatments.

## 4. Results and discussion

### 4.1. Preparation of ETO/FAM RS

The design results proposed 16 different ETO/FAM RS, each was prepared separately with the incorporation of 60 mg/10 ml ETO and 20 mg/10 ml FAM (Table 2). By visual inspection and through the preparation process, the formulated RS exhibited fluid behavior suggesting an ease of swallowing. Further, a superlative Na citrate concentration of 0.25% w/v was incorporated as reported by Bhavsar et al., to perpetuate the fluidity of prepared RS before the administration and enhance the gelation post-administration (Bhavsar, 2012). Likewise, calcium carbonate quantities were used as a calcium exporter and as a gas-liberating compound to generate carbon dioxide and entrap it in the gelling network matrix leading to the floating of the RS.

The grounds for incorporating KGL in the floating raft formulations are abundant. It exhibits significant biocompatibility, biodegradability, hydrophilicity, non-toxicity, and low-cost features (Yu et al., 2006). It is excessively used as a thickening and coating agent in the food industry. It serves as a biomedical material as well (Liu et al., 2004). It is suggested that KGL can be used as a carrier for oral controlled drug delivery by some modifications with physicochemical methods (Yu et al., 2006). The results of a study conducted by Alvarez-Manceñido et al., pointed out the potentiality of KGL to develop delivery formulations capable of maintaining physical integrity and controlled drug release for up to an 8 h period (Alvarez-Manceñido et al., 2008). During the preparation process, it was noticed that the raft consistency was improved after the addition of KGL due to its thickening ability. Reaching an equilibrium between the system consistency for controlled drug release and the outpouring ability of the raft was significantly achieved.

Pr incorporation is substantial for improving the gelation mechanism and prolonging the drug release, with no impact on the floating and influx attributes. It plays an essential role in the buoyancy of the prepared systems because of their low density (Hamdani et al., 2002). Other reported advantages are their amphiphilicity which enables consistency with polymeric aqueous-based systems and their capability to extend drug release from several pharmaceutical preparations (J. C. Shah et al., 2001). The homogenization technique results in a good texture and homogenous emulsion based on the lipid emulsifying ability. The flawless formulae must exhibit reasonable portability, floating competencies (floating speed and period), enhanced drug-controlled release, and the ability to create a robust gel with mechanical strength strong enough to oppose pressure throughout stomach contractions.

### 4.2. In vitro characterization of ETO/FAM RS

#### 4.2.1. Factorial design analysis

To scrutinize the role of the studied factors on the various characteristics of the drug delivery systems prepared, a 24-full factorial design was employed. Six responses; GLT (Y_1_), FLT (Y_2_), the percent of ETO released after 1 h (Y_3_) and 8 h (Y_5_), and the percent of FAM released after 1 h (Y_4_) and 8 h (Y_6_) were investigated and evaluated solely. The sixteen experimental systems and their detected responses are displayed in Table 2. The coded factors for each response, are used as the predictive model for interpretation purposes and are represented in empirical equations. In these equations, the coefficient values and signs are associated with the effect of our variables on the targeted responses. The coefficient’s positive sign denotes a synergistic action, and the negative sign resembles an antagonistic action on the response. The larger number of coefficients proposes a more potent influence of the independent variable on the response. There weren’t any data transformations proposed by Box-Cox plots for all the responses. To standardize the significant level of the analyzed factors in each response ANOVA test was used. High R^2^ value numbers might designate a sturdy connection between the predicted and the experimental values. As well, an adequate precision of more than 4 indicates a high signal-to-noise ratio, i.e. adequate signal (Muthukumar et al., 2003).

#### 4.2.2. Gelation study

The gelling manner is a crucial parameter in the optimization of floating raft-forming systems concerning oral intake and drug delivery control. The rigid gelation is maintained into a contact gel patch in the acidic media (pH 1.2) which is critical for controlled drug release. Hence, the gelation study was performed in 0.1 N HCl (pH 1.2). The observed gels of the formulations were rigid for more than 12 hours. The empirical equation representing the effect of factors on the gelation lag time response is stated below:

(2) $$Y_{1}=\;+51.91\;-{11.42\;X}_{1}-{8.47\;X}_{2}-{9.55\;X}_{3}+{0.28\;X}_{4}+6.20{\;X}_{1}X_{2}-{3.22\;X}_{1}X_{3}+{7.61\;X}_{1}X_{4}+{5.08\;X}_{2}X_{3}-{0.35\;X}_{2}X_{4}-{9.39\;X}_{3}X_{4}\left(R^{2}=\;0.982\right)$$

LM-pectin and gellan gum were two different types of hydrogel enhancers that were used to evaluate the gelling ability of RS formulations. On the physical observation of the prepared systems, LM-pectin-based systems showed a satisfactory gel strength with a favorable viscosity compared to the gellan-based systems. The fluid-like behavior of gellan gum at small concentrations that are utilized for the preparation of fluid gels played the main role in obtaining a weak viscous liquid gel that won’t be able to resist the peristaltic movements in the stomach (Jaroenkietkajorn et al., 2018; Saha & Bhattacharya, 2010). Although the gelation lag time was minimum as required, the poor gel strength and low viscosity would negatively impact the rigidity of the formula.

In the former equation, the main effect of the type of hydrogel enhancer (X_1_); LM-pectin provides the largest significant effect with a coefficient of (-11.42) on the GLT response (Y_1_). The regression coefficients of all effects and interactions were negative (antagonistic effect) except the interactions (X_1_X_4_ and X_2_X_3_) were positive (synergistic effect). The concentration of calcium carbonates (X_4_) showed an insignificant effect on the gelation lag time.

Preliminary studies encourage the use of 1% of Na alginate and 0.1% of hydrogel enhancer (LM-pectin or gellan gum) that would enhance the gel formation in various mechanisms. Generally, the gelation mechanism involves the development of the junction zones which is the assembly of the disseminated polymer segments in a definite way to create a three-dimensional cubic (Saha & Bhattacharya, 2010). Alginates form the gel through the ionotropic mechanism. Ionotropic gelation takes place via cross-linking of hydrocolloid chains with ions either by diffusion or internal gelation, typically a cation-mediated gelation process of negatively charged polysaccharides (Phillips & Williams, 2009). It is worth noting that hydrogels are developed in the existence of divalent cations such as Ca^2+^, so calcium carbonates are utilized in the systems to act as an insoluble dispersion liberating calcium ions. The mechanism of LM-pectin gelation is related to the “egg-box” mechanism describing the potential LM-pectin and calcium ion interactions due to their similar structures and crosslinking behavior. In the “egg-box” mechanism, junction zones are structured by binding between Ca^2+^ and non-esterified galacturonic acid units. Calcium ions interact with polygalacturonate chains through the oxygen atoms found in the carboxylate group or the glycosidic bond. Furthermore, the acidic pH facilitates gel formation via hydrophobic interactions and the formation of hydrogen bonds (Gawkowska et al., 2018). While in the case of gellan gum systems, gelation occurs by aggregation of double helices. So, in the presence of calcium ions, aggregation and consequent gelation between gellan gum and calcium ions occur by direct site-binding of the divalent ions between gellan gum double helices. The high concentrations of acid cause excessive aggregation and a consequent reduction in gel strength (Morris et al., 2012). This clarifies why the raft systems containing gellan gum show poor gel strength on physical observation.

The antagonistic effect of KGL may be explained by the fact that the addition of KGL facilitates gel formation through the heat set gels mechanism, which requires the application of heat to the gel. This mechanism is occurred by diversification and subsequent arrangement of the molecules within the network. Thus, KGL forms a highly viscous dispersion gel with enough strength to encounter the shear forces in the peristaltic movements in the stomach. The Na citrate (0.25% w/v) incorporated in the raft formulations was ideal in preventing any early gelation as a chelating agent that congregates the free Ca^2+^ ions and ensures gelation in the stomach acidic medium (M. H. Teaima et al., 2020).

Previously, the addition of lipids like Pr was known for their biodegradability, as a gelling agent (DEVI & Agarwal, 2019), and their ability to improve gelling capacity (Abou Youssef et al., 2015). The exact role of Pr in improving gelling ability and decreasing the gelation lag time is not clear. However, it is suggested that the Pr’s gelling ability contributes to its hydrophobic nature as it is a mixture of different triglyceride types, lipophilic matrix, and s low-density behavior (M. H. Teaima et al., 2020). These factors ameliorated how the increase in the concentration of Pr could decrease the GLT of the prepared RS.

#### 4.2.3. Floating assessment

The total floating time and the floating lag time are the two parameters evaluated to study the buoyancy ability of the raft systems. The 16 formulations showed appropriate total floating time, each one maintained a total floating time of more than 12 h. The following statistical equation describes the independent variables affecting FLT:

(3) $$Y_{2}=\;+18.48\;-{2.94\;X}_{1}-{10.77\;X}_{2}+{2.13\;X}_{3}-{3.73\;X}_{4}+{5.48\;X}_{1}X_{2}+{1.16\;X}_{1}X_{3}+{3.38\;X}_{1}X_{4}-{3.34\;X}_{2}X_{3}+{4.53\;X}_{2}X_{4}-{1.02\;X}_{3}X_{4}\left(R^{2}=\;0.984\right)$$

The equation revealed that the concentration of the KGL provides the largest potent antagonistic effect on the floating lag time with a coefficient of (-10.77). This is attributed to the main role of KGL on the floating properties of raft systems. As the concentration of KGL increased, the floating lag time decreased because the swelling behavior improved thus forming a strong viscous matrix structure that is rigid enough to entrap the gas bubble when coming in contact with the water (Mostafavi et al., 2011).

Another factor attributed to the buoyancy of floating raft systems is the use of calcium carbonate as a gas-forming agent, in the acidic stomach media, a sufficient amount of carbon dioxide bubbles is rapidly produced and entrapped in a gel network resulting in buoyancy enhancement of the formulation and triggering its upward movement (M. H. Teaima et al., 2020). The minimized floating lag time would protect the formulation from the peristaltic movement, ensuring the floating of the raft and hence, improving the sustained release of both drugs in the stomach.

#### 4.2.4. In vitro release studies

**Figure 1.**
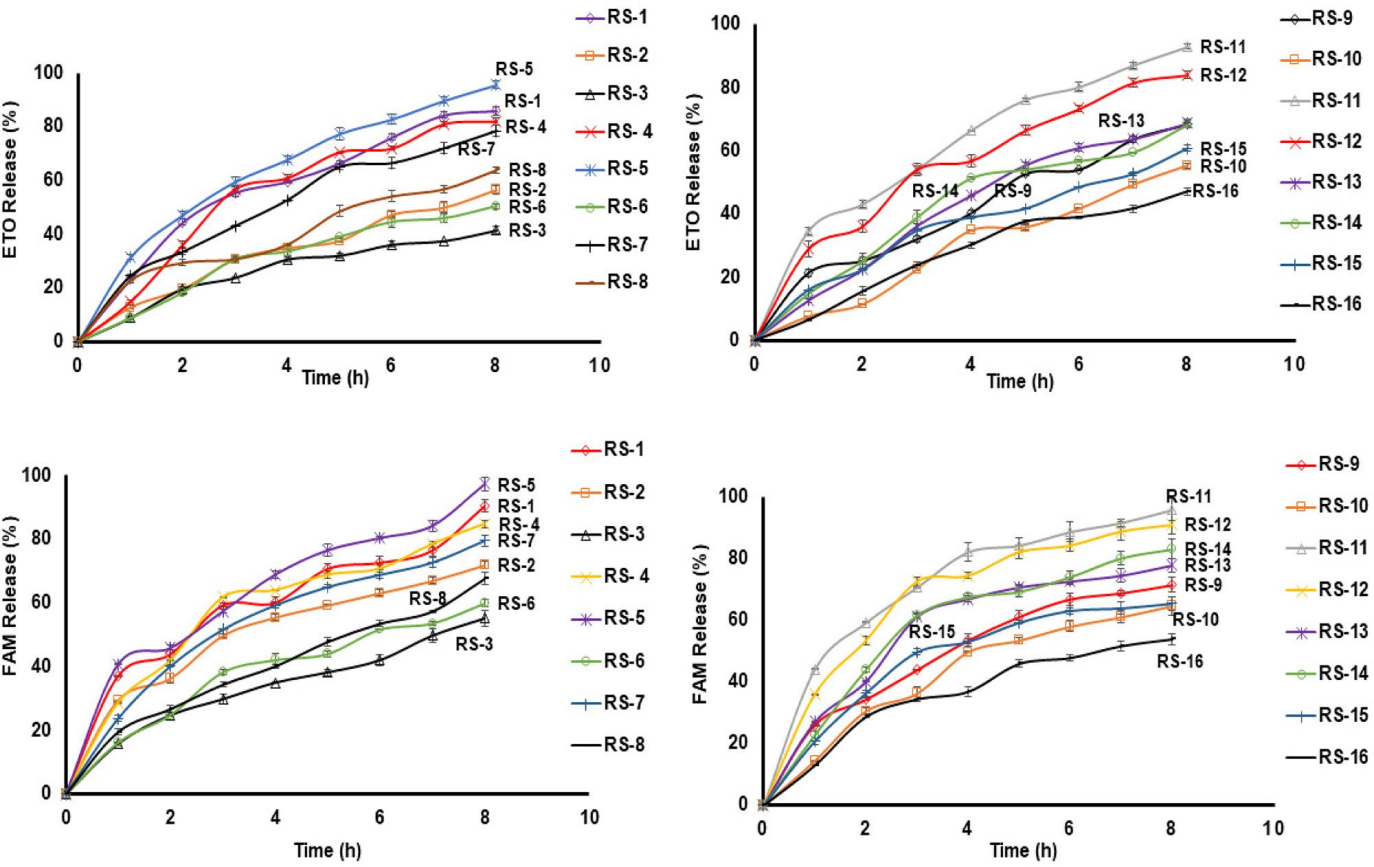
Dissolution Profiles of ETO/FAM release from different gastroretentive RS formulations. Each point represents the mean value ± standard deviation (*n* = 3).

The dissolution vessels were filled with 900 ml of 0.1 N HCL and the device’s temperature was adjusted at 37 ± 0.1 °C for the release conduction with 50 rpm, which was slow enough to avoid any disturbance to the formulations (M. H. Teaima et al., 2020). The main goal was to design a superior release effect for the FAM drug to act as a barrier from ETO for stomach protection followed by the ETO release and then maintaining a successful controlled release for both drugs. This strategy will allow significant protection from the common side effect of analgesic drugs, GIT disturbances, and ulcers. Figure 1 represents the dissolution profile of the RS formulations.

##### 4.2.4.1. In vitro release after 1 h

The pKa of each drug can control its release in the first hour when it encounters the acidic pH. It was reported that ETO and FAM exhibit pKa 4.5 and 6.98 (Das et al., 2011; Islam & Narurkar, 2011) and aqueous solubility of 0.0245 mg/ml and 1.353 mg/ml, respectively (Mummaneni & Vasavada, 1990; Sapkal et al., 2020). The higher pKa, the weaker the acid, the weak acid tends to have a strong conjugate base that will increase its solubility in the acidic media. It is believed that the minimal difference between the pKa of the two studied drugs served the benefit of the superior release of FAM over the ETO release to protect the GIT. On the other hand, the solubility of FAM is slightly higher than that of ETO which might act as a contributing factor in the earliest release of FAM. The equations representing the release of ETO after 1 h (Y_3_) and FAM (Y_5_) are mentioned below:

(4) $$Y_{3}=\;+18.13\;-0.30{\;X}_{1}-{4.17\;X}_{2}-{3.57\;X}_{3}-{4.47\;X}_{4}+{2.92\;X}_{1}X_{2}-{1.38\;X}_{1}X_{3}-{2.97\;X}_{1}X_{4}-0.{34\;X}_{2}X_{3}+{1.25\;X}_{2}X_{4}+{1.33X}_{3}X_{4}\left(R^{2}=\;0.995\right)$$

(5) $$Y_{5}=+26.04-0.46X_{1}-3.13X_{2}-7.29X_{3}-3.57X_{4}+0.74X_{1}X_{2}+0.33X_{1}X_{3}-2.66X_{1}X_{4}+0.87X_{2}X_{3}+0.98X_{2}X_{4}+0.57X_{3}X_{4}(R^{2}=0.993)$$

In both equations for both drugs, KGL concentration, conc. of Pr (X_3_), and CaCO_3_ conc. (X_4_) provided an antagonistic influence on the first hour of drug release response (Y_3_). While in equation 3, the sign of the interactions X_1_X_2_ was positive (synergistic effect) on drug released percentage after 1 hour. Otherwise, equation 4 revealed that all the interactions were insignificant to the release of FAM after 1 h.

##### 4.2.4.2. In vitro release after 8 h

The release of the drugs after 8 h has common synergistic and antagonistic effects. Equations 5 and 6 resemble the former equations in the significant antagonistic effect of KGL concentration, conc. of Pr, and CaCO_3_ conc. representing the drugs’ release after eight hours as follows:

(6) $$Y_{4}=\;+68.77\;-0.{55X}_{1}-{7.41X}_{2}-{10.53\;X}_{3}-{7.82\;X}_{4}+{4.29\;X}_{1}X_{2}+0.{23\;X}_{1}X_{3}-0.50{\;X}_{1}X_{4}-0.{88\;X}_{2}X_{3}-0.{14\;X}_{2}X_{4}+{1.56\;X}_{3}X_{4}\left(R^{2}=\;0.978\right)$$

(7) $$Y_{6}=\;+75.71\;-0.{15X}_{1}-5.00X_{2}-{10.92\;X}_{3}-5.0{5\;X}_{4}+{2.91\;X}_{1}X_{2}-0.{64\;X}_{1}X_{3}-0.{52\;X}_{1}X_{4}-{1.16\;X}_{2}X_{3}+0.40{\;X}_{2}X_{4}+0.{68\;X}_{3}X_{4}\left(R^{2}=\;0.977\right)$$

In terms of equations 5 and 6, all the interactions were insignificant to the release of the drug after 8 h except for interaction X_1_X_2_ which has a positive sign indicating a synergistic effect on the drug’s release.

According to different studies, KGL has a synergistic gel effect when combined with other polysaccharides (alginates, LM-pectin). These interactions can yield strong and thermoreversible hydrogels (Alvarez-Manceñido et al., 2008; Brenner et al., 2015). As well, KGL can form a highly viscous rigid gel that can provide a strong protective matrix for successful controlled drug release. Drug diffusion and ion-ion interaction are two potential mechanisms for sustained drug delivery. The size of the diffusing molecule is an important factor in controlling the release. Both drugs are considered small molecules and the mechanism of controlled drug release of small molecules was explained by Alvarez-Manceñido et al., that either more entanglement points or a stronger gel network enhanced the control over drug release by crowding or sieving mechanism (Alvarez-Manceñido et al., 2008).

As an amphiphilic lipid, Pr can control ETO/FAM delivery by both the leisurely erosion of matrix and drug diffusion mechanisms. As in the gastric area, they can be reorganized into lipid bilayers and form a reversed micellar phase (cubic phase). The Cubic phase has a unique structure and resemblance to biomembranes and possesses remarkable stiffness and high viscosity that maintained its ability to incorporate and control the release of different drug molecules by slowing the drug’s diffusion (J. C. Shah et al., 2001). The leverage of incorporation of a lipophilic polymer, bolstered the lipophilicity of the raft matrix system thus minifying the penetration of buffer molecules into the formulations and hence, decreasing the diffusion of the drug from the matrix. The concentration of CaCO_3_ affects ETO/FAM release rate and extent too as it improves the gelation capacity and hence forms a denser matrix through which the drugs diffuse to the media from the raft formulations.

#### 4.2.5. Kinetic modeling on drug release

The best fit kinetic paradigm was selected based on the most convenient model exemplified by the identification of the coefficient (R^2^); its value is near 1. For the ETO delivery, Korsmeyer-Peppas was the best fit for all raft systems except for RS-12, RS-13, and RS-14 which exhibit First-order drug delivery behavior. In the case of the FAM delivery profile, Korsmeyer-Peppas was also the best model for all formulations except for RS-12 and RS-14 which showed Higuchi diffusion (Table 3).

**Table 3.** Release kinetics pattern for different ETO/FAM RS.

Code of formula	ETO					FAM				
	Zero-order	First-order	Higuchi model	Korsmeyer–Peppas model		Zero-order	First-order	Higuchi model	Korsmeyer–Peppas model	
	R^2^	R^2^	R^2^	R^2^	n	R^2^	R^2^	R^2^	R^2^	n
RS-1	0.852	0.980	0.979	0.985	0.575	0.405	0.943	0.978	0.992	0.349
RS-2	0.927	0.983	0.961	0.990	0.681	0.578	0.95	0.995	0.991	0.420
RS-3	0.983	0.981	0.860	0.983	0.971	0.491	0.96	0.988	0.986	0.548
RS-4	0.840	0.988	0.984	0.999	0.561	0.648	0.962	0.996	0.989	0.365
RS-5	0.919	0.983	0.972	0.994	0.657	0.549	0.946	0.988	0.995	0.476
RS-6	0.892	0.967	0.967	0.984	0.633	0.597	0.94	0.997	0.989	0.558
RS-7	0.888	0.991	0.982	0.994	0.609	0.566	0.95	0.993	0.997	0.472
RS-8	0.789	0.891	0.956	0.956	0.519	0.614	0.939	0.998	0.998	0.618
RS-9	0.950	0.965	0.916	0.972	0.786	0.587	0.882	0.987	0.992	0.498
RS-10	0.982	0.975	0.865	0.983	0.960	0.496	0.814	0.981	0.989	0.601
RS-11	0.592	0.977	0.975	0.996	0.385	0.558	0.854	0.99	0.995	0.332
RS-12	0.850	0.992	0.982	0.988	0.574	0.547	0.832	0.985	0.981	0.386
RS-13	0.937	0.985	0.918	0.970	0.758	0.572	0.838	0.99	0.950	0.428
RS-14	0.897	0.986	0.964	0.983	0.641	0.54	0.782	0.989	0.975	0.507
RS-15	0.898	0.970	0.976	0.991	0.626	0.607	0.852	0.995	0.978	0.452
RS-16	0.955	0.980	0.979	0.980	0.781	0.598	0.809	0.994	0.981	0.478

Abbreviations: R^2^, regression coefficients; n, release exponent.

The release exponent (n) value was computed to describe the delivery as either Fickian diffusion *n* ≤ 0.5, anomalous diffusion (non-Fickian), or 0.5< *n* < 1. The “n” values for the ETO delivery were more than 0.5 suggesting a non-Fickian diffusion except for RS-11. While the “n” values for the FAM delivery were less than 0.5 specifying a Fickian diffusion except for RS-3, RS-6, RS-8, RS-10, and RS-14 show non-Fickian diffusion. So, the delivery mechanism of FAM from RS is controlled by diffusion through channels existing in the structure of the hydrogel. But, the release of ETO from raft systems in acidic pH (1.2) is managed by a combination of erosion and diffusion of polymers used in raft systems (K. U. Shah & Khan, 2012; Arfan et al., 2022).

#### 4.2.6. Viscosity measurement

In terms of viscosity, an effective floating raft system would exhibit several features. Firstly, it should achieve optimum viscosity that permits easy swallowing. Secondly, it would exhibit a quick liquid-gel conversion and float when it comes in contact with stomach fluids. Finally, the in-situ gel must preserve its integrity and frustrate any dissolving or eroding action for a long time especially during buoyancy to perpetuate the sustained-drug release.

KGL exhibits shear-thinning behavior in which viscosity decreases with increasing the shear rate. This would be beneficial in the patient’s swallowing ease and during the preparation process. KGL molecule includes glucose and mannose that there are loaded with hydroxyls. These hydroxyls develop hydrogen bonding in the KGL solution with the presence of extra hydrogen bonding in the molecules that increases the viscosity. During the shearing, these hydrogen bonds broke down and the solution viscosity lessened because the KGL molecular series extend along the direction of shear force under the action of shear force. The highest concentration of KGL (0.8% w/v), produces more viscosity and better shear thinning behavior and this may be due to that at low concentrations of KGL (0.5% w/v), its molecules in the aqueous media are present as trackless wire clusters and can produce intermolecular entanglements that produce less viscous sol (W. Zhang et al., 2022).

**Figure 2.**
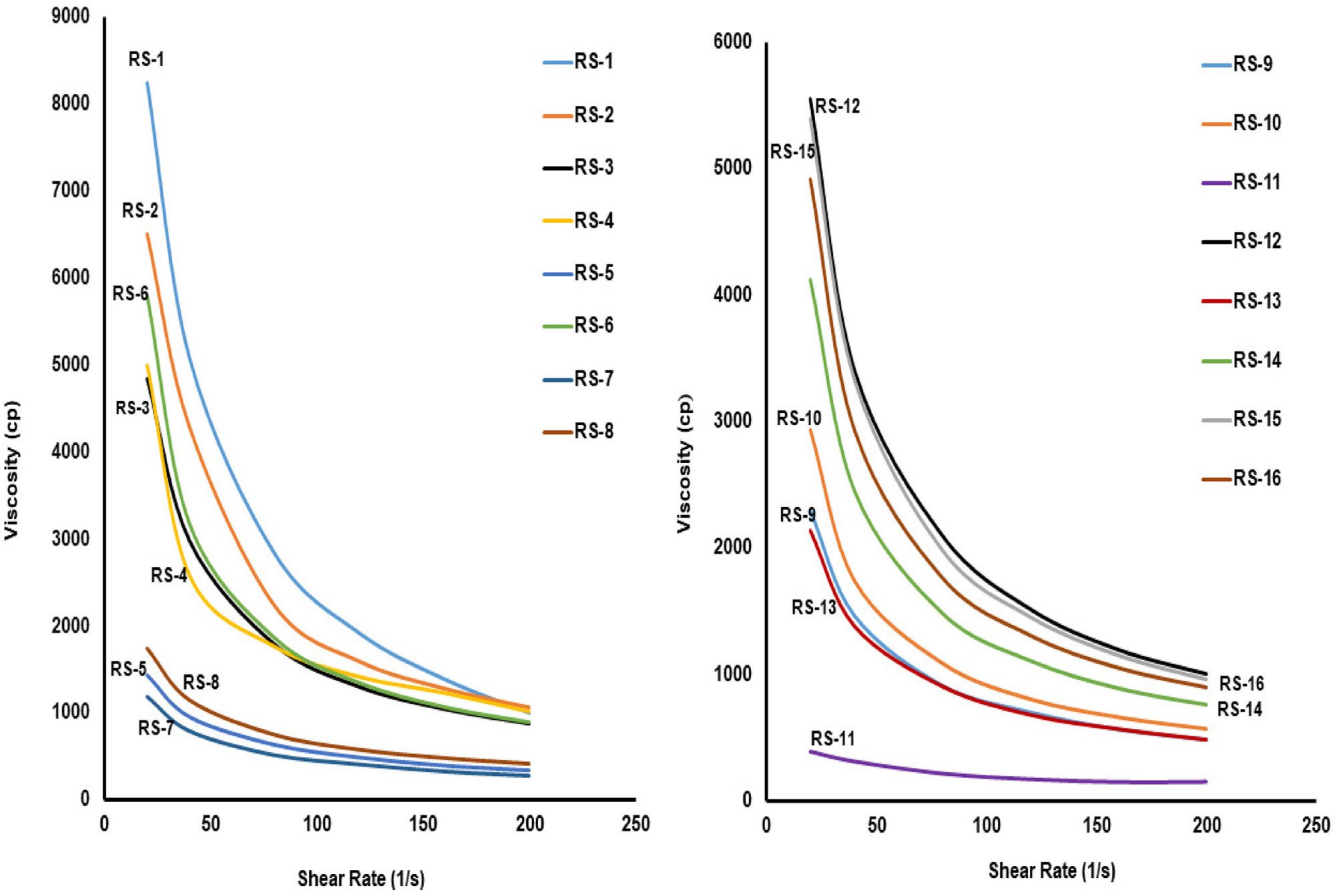
Viscosity flow curves of ETO/FAM RS formulations.

Divalent metal ions help both Na alginate and LM-pectin to transform the solution into a gel. For instance, the divalent cations (ca^+2^ ions) have an internal ionotropic gelling potentiality on the gel-forming polymers rendering them excellent in promoting gelation. Further, the gel-forming polymer can compose double helices connected by Van der Waals attraction. Few of these helices may combine with cation-mediated clusters, that will inter-link the polymers and create the egg-box configuration which augments the gel network strength and is then subjected to a fast liquid-gel transformation (Abou Youssef et al., 2015; M. H. Teaima et al., 2020). Table 4 and Figure 2 illustrate the rheological data of the prepared ETO/FAM RS.

**Table 4.** Viscosity data results of the ETO/FAM RS.

Code of formula	Rheological data	
	Farrow´s constant (N)	Flow behavior
RS-1	0.9573	Pseudoplastic
RS-2	0.8517	Pseudoplastic
RS-3	0.8977	Pseudoplastic
RS-4	0.7384	Pseudoplastic
RS-5	1.1452	Pseudoplastic
RS-6	0.8841	Pseudoplastic
RS-7	1.2043	Pseudoplastic
RS-8	1.0915	Pseudoplastic
RS-9	1.0394	Pseudoplastic
RS-10	0.9968	Pseudoplastic
RS-11	1.5584	Pseudoplastic
RS-12	0.7311	Pseudoplastic
RS-13	1.0448	Pseudoplastic
RS-14	0.9294	Pseudoplastic
RS-15	0.8786	Pseudoplastic
RS-16	0.8973	Pseudoplastic

Abbreviations: R^2^, regression coefficients; n, release exponent.

#### 4.2.7. Nomination of optimized raft system (ORS)

**Figure 3.**
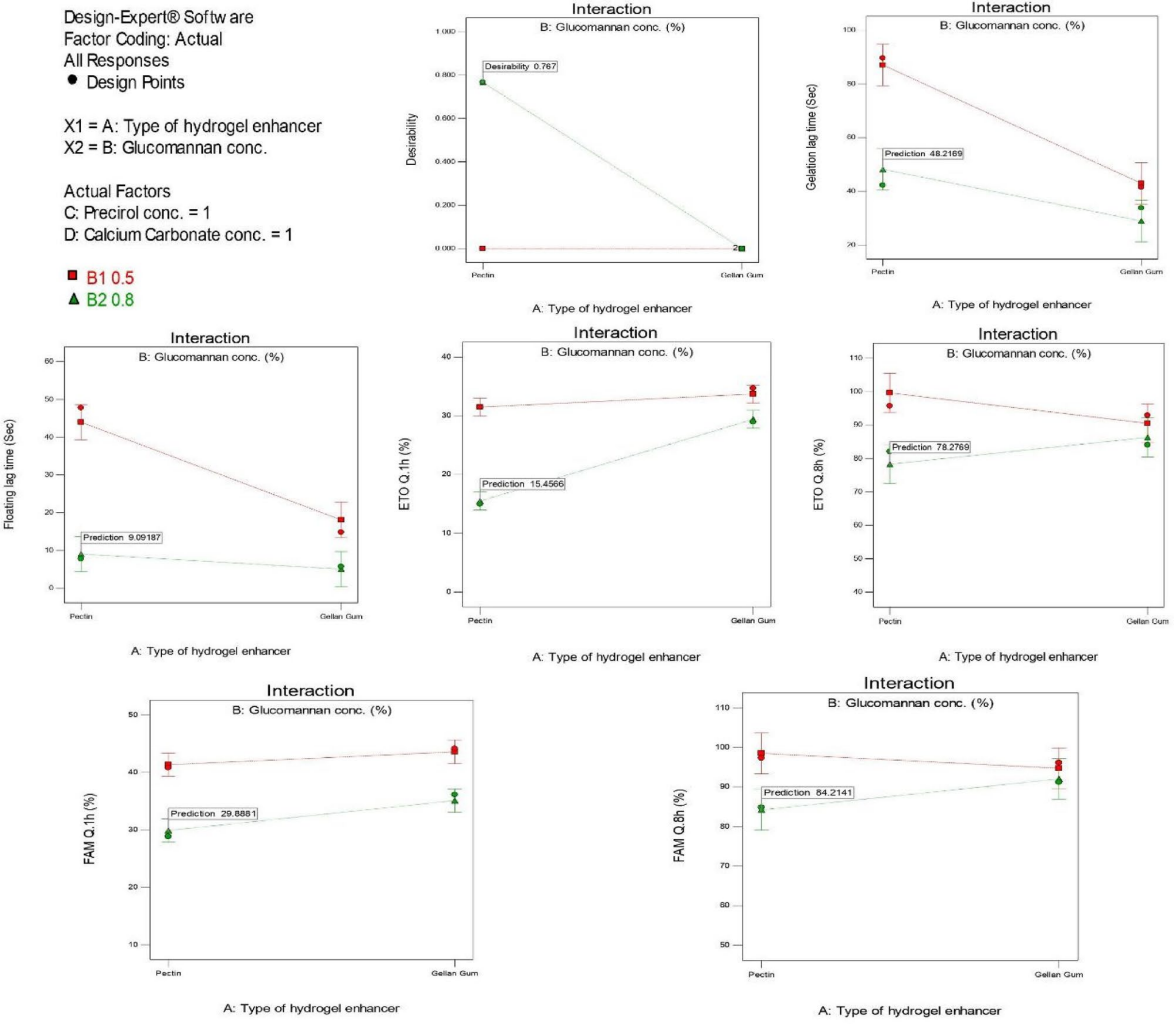
Response interaction plots for the effect of significant formulation variables on the simultaneous prediction of all responses; Gelation lag time (a), Floating lag time (b), ETO release % after 1 h (c), and FAM release % after 1 h (d), ETO release % after 8 h (e), and FAM release % after 8 h (f).

The Design Expert^®^ program applied the constraints and selected the optimum system through the desirability function. The main target was to manage the drug delivery with the least GLT and FLT. The program nominated the RS-4 formula as the best RS formula with a desirability of 0.769 and hence, RS-4 was examined for stability and in vivo evaluation. The interactions between the possible effect of all factors on the different responses are represented in Figure 3.

#### 4.2.8. Short-term stability study

The stored samples of the raft system showed no considerable aggregation or alteration in their physical appearance, floating behavior, or the drug released after 45 and 90 days at both 4 °C and 25 °C (Srinivas & Sagar, 2022). However, a slight increase in viscosity was observed upon storage, but it didn’t affect the pourability (Abou Youssef et al., 2015). The values of GLT, FLT, and ETO release percentage after 1 and 8 h, as well as, FAM release percentage after 1 and 8 h for both the fresh and the stored ORS are shown in Table 5. The studied responses had no significant change (*p* > 0.05 for all values).4.2.9 Differential Scanning Calorimetry

**Table 5.** Effect of storage conditions on the physical properties of optimum ETO/FAM RS after time intervals (45 and 90 days) at both 4 and 25 ± 3 °C.

Parameter	Fresh ORS	ORS after 45 days at 4 °C	ORS after 45 days at 25 °C	ORS after 90 days at 4 °C	ORS after 90 days at 25 °C
GLT (sec)	42.29 ± 0.38	41.78 ± 0.41	40.07 ± 0.07	40.51 ± 0.54	39.14 ± 0.21
FLT (sec)	7.81 ± 0.67	7.08 ± 0.21	6.74 ± 0.08	6.14 ± 0.49	8.12 ± 0.16
ETO percentage released after 1 h (%)	14.94 ± 0.67	15.35 ± 0.38	15.12 ± 0.89	13.48 ± 1.07	14.09 ± 0.85
FAM percentage released after 1 h (%)	28.83 ± 0.21	30.41 ± 0.84	29.01 ± 1.09	25.84 ± 1.01	26.11 ± 0.91
ETO percentage released after 8 h (%)	82.04 ± 1.29	81.54 ± 1.51	80.32 ± 1.71	79.05 ± 2.11	79.43 ± 1.95
FAM percentage released after 8 h (%)	84.84 ± 1.11	84.02 ± 1.39	82.61 ± 2.08	80.54 ± 1.89	83.51 ± 2.18

Data presented as mean ± SD (*n* = 3).

Abbreviations: ORS, optimum raft system; GLT, gelation lag time; FLT, floating lag time.

#### 4.2.9. Differential scanning calorimetry

**Figure 4.**
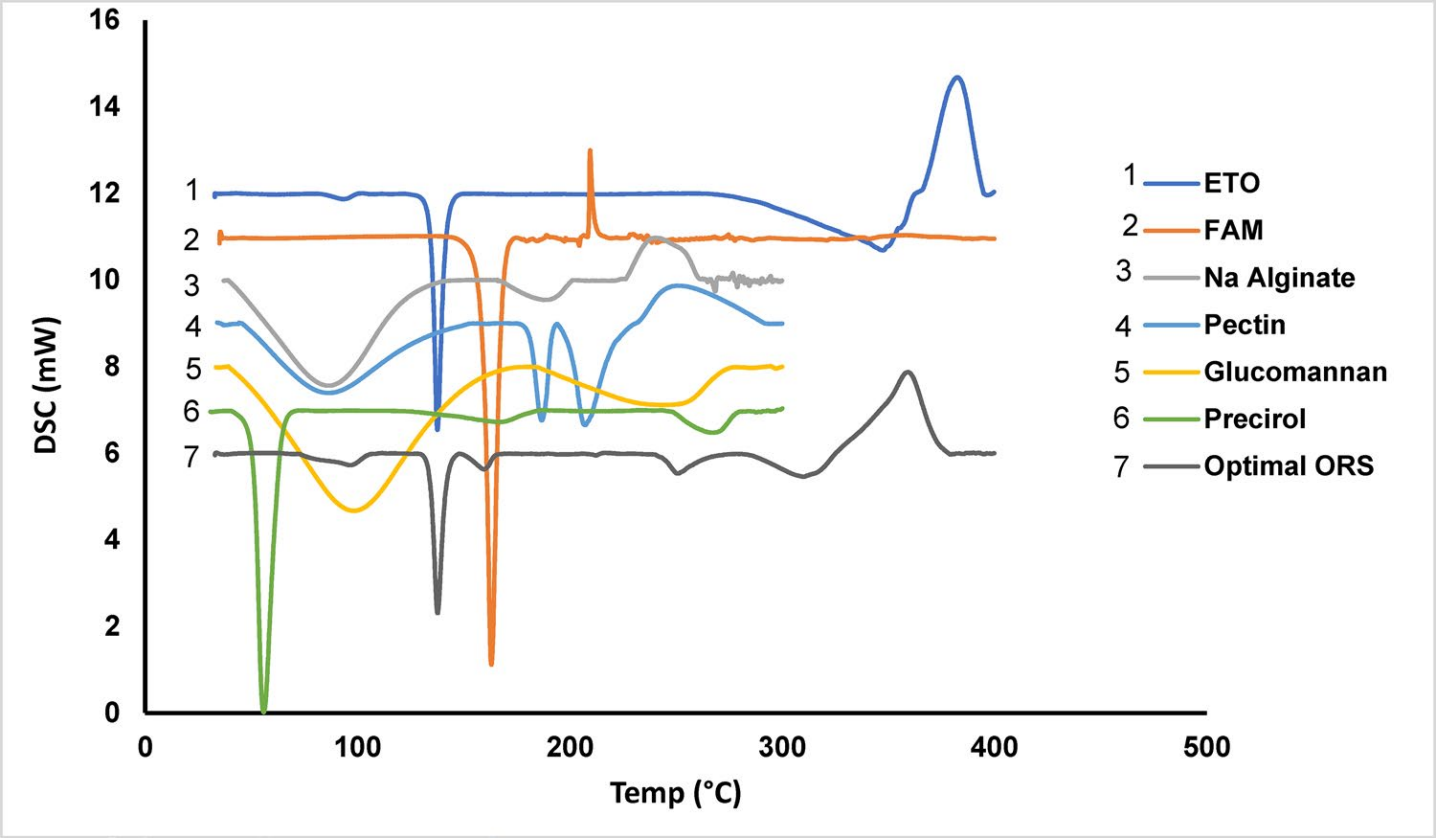
DSC thermograms of pure ETO, FAM, KGL, Precirol®, pectin, Na alginate, and the physical mixture of optimized raft system (ORS).

DSC is contributed to the evaluation of two important features of the powders used in the formula; the melting behavior of the excipients and the drug’s crystallinity or amorphous nature within the developed formulations. The DSC thermogram of both drugs ETO and FAM and all the materials used in the optimum formula (Figure 4). ETO has one distinct endothermic peak at 137.59 °C and another prominent exothermic peak at 382.4 °C while FAM exhibits an endothermic peak at 162.98 °C and another exothermic one at 209.47 °C. The endothermic peak of ETO represents its melting behavior and its highly crystalline nature (Das et al., 2011). The endothermic peak of FAM resembles its melting point and non-hygroscopic crystalline nature (Ain et al., 2017).

The DSC analysis results of the KGL powder showed endothermic peaks at 97.84 and 244.11 °C. The first endothermic peak is probably related to the water’s evaporation and the second could be due to the thermal decomposition of KGL. This could be explained by the fact that there are two kinds of crystals in KGL; one is a KGL-water crystallite and the other is a KGL-KGL crystallite in which both are formed by hydrogen bonds (Li et al., 2005; S.-Q. Wang et al., 2017). Also, the Precirol^®^ thermogram displays a single sharp endothermic peak at around 55.7 °C, representing its melting point. The endothermic of peak 186.66 °C of LM-pectin indicated its melting point while the 207.29 °C peak explains the existence of water molecules in it, the availability of hydrogen bonding between the galacturonic acid units, and conformational alterations in the galacturonan ring (Dranca & Oroian, 2019).

The endothermic peak of ETO is shown in the thermogram of ORS indicating that there is no interference between ETO and excipients used in the formula and that the drug is still in its crystalline form. Additionally, the peaks of ETO and FAM in the physical mixture were slightly shifted which might indicate that parts of the drugs had lost their crystallinity while the other parts remained intact during the DSC scan (Abouelatta et al., 2018). On the other hand, Na alginate exhibit two endothermic peaks (86.13 °C and 188.45 °C) the former one is ascribed to the water loss of its lipophobic polymer groups and the latter one represents its melting temperature. The exothermic peak at 238.5 °C is contributed to the degradation of the polymer (P. R et al., 2013).

#### 4.2.10. Fourier transform infrared spectroscopy

**Figure 5.**
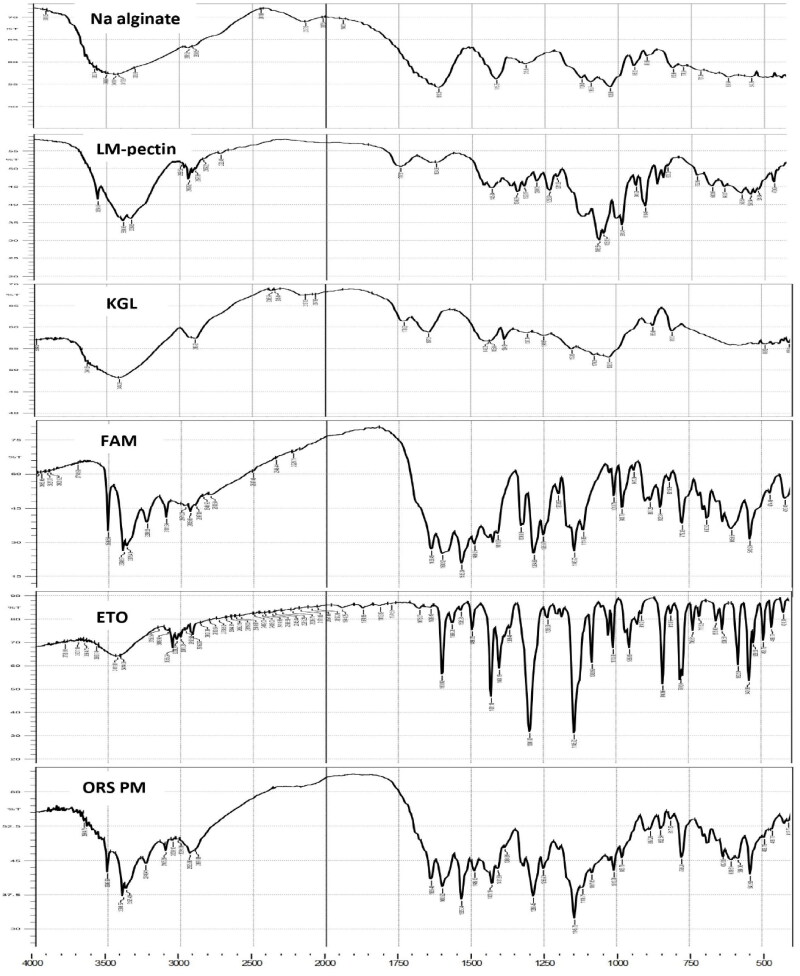
FT-IR spectra of pure ETO, FAM, Na alginate, pectin, and KGL and the physical mixture of optimized raft system (ORS).

FTIR spectroscopy analysis was conducted to analyze physicochemical interactions between the drugs (ETO/FAM) and excipients used in ORS. FTIR spectra of pure ETO, FAM, KGL, LM-pectin, and physical mixture are shown in Figure 5. The FTIR spectra of ETO showed characteristic peaks at 1600.92 cm^−1^ (C = N stretching vibration), 1431.18, 1300.02, 1145.72, and 1083.9 cm^−1^ (S = O stretching vibration), and 840.96, 779.24, and 636.51 cm^−1^ (C–Cl stretching vibration) (Das et al., 2011). As well, the main absorption bands of FAM are at 3506.59 and 3398.57 cm^−1^ (N-H stretching of amide guanidine and sulfo groups), 3375, 3236, and 3101.54 cm^−1^ (C-H stretching), 1639.49 cm^−1^ (C = N stretching), 1600.92 and 1535.34 cm^−1^ (N-H bending), and 1114.86 cm^−1^ (S-O stretching) (Rahamathulla et al., 2021).

FTIR spectra of pure KGL powder showed a strong absorption peak at 3414 cm^−1^ (stretching vibration of –OH). The peak appeared at 2893.22 cm^−1^ (stretching vibrations in the –CH_2_ or –CH_3_ groups), 1728.22 cm^−1^ (stretching vibration of the carbonyl-acetyl groups of KGL molecules), 1647.21 cm^−1^ (CeO-OH stretching vibrations), and the absorption peak at 1026.13 cm^−1^ (CeOeC vibrations) (L.-X. Wang et al., 2020). For pectin, the characteristic peaks appeared at around 3390.86 cm^−1^ (stretching of –OH groups), 2939.5 cm^−1^ (C–H stretching vibration), and 991.4 cm^−1^ (–CH–O–CH– stretching). The peak at 1,068.5 cm^−1^ suggested the presence of (–CH–OH) in aliphatic cyclic secondary alcohol (Mishra et al., 2008). The characteristics peaks of the drugs that appeared in the FTIR spectrum of the optimum formula indicate that there are no changes in the chemical structure that occurred during the formulation process. Additionally, it indicates that there are no drug-excipient interactions took place. So, there are no physical or chemical interactions of ETO, FAM with KGL, or any other additive excipient used in the ORS.

### 4.3. In vivo X-ray study in human volunteers

**Figure 6.**
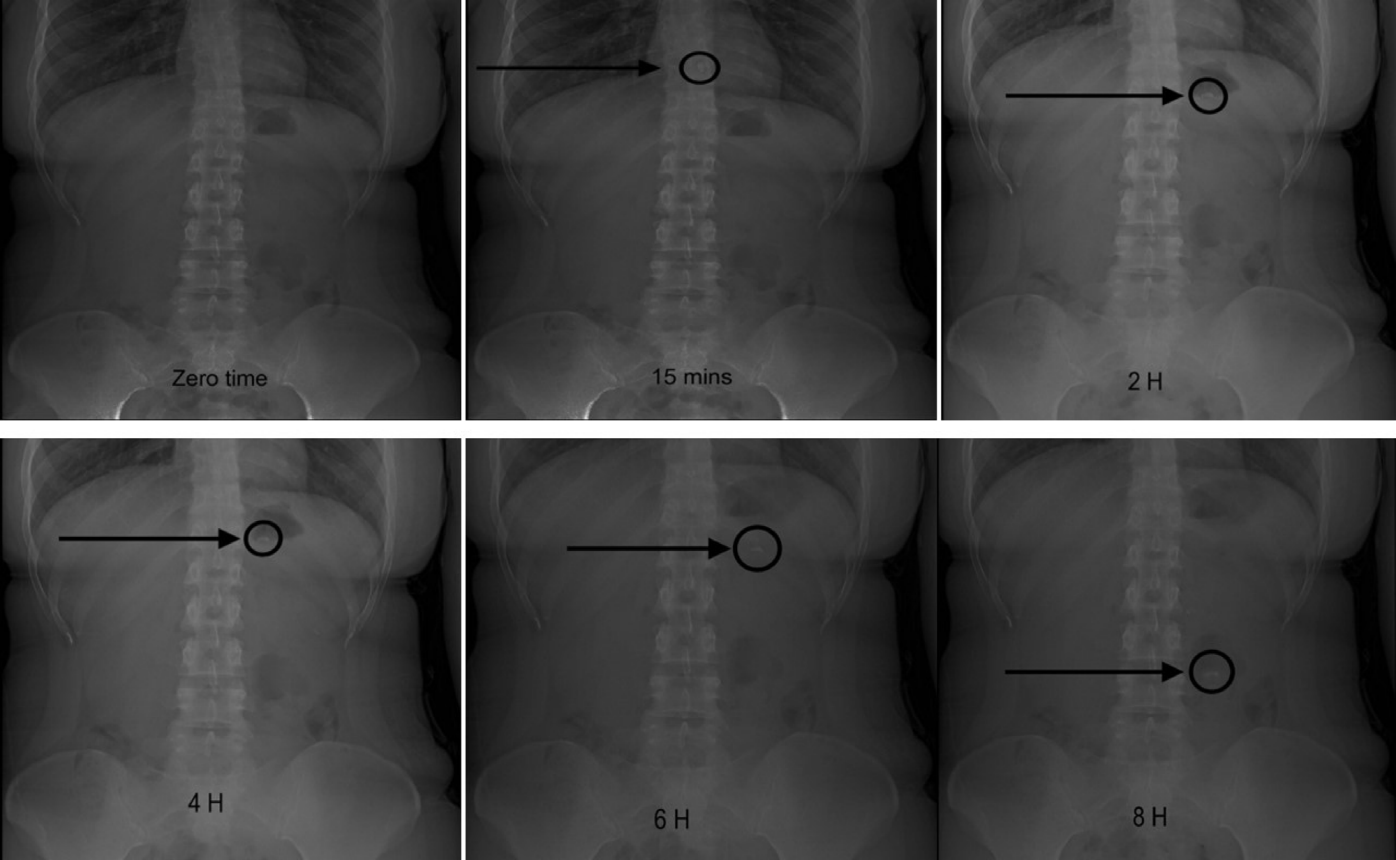
X-ray images of the ORS (BaSO_4_-loaded) representing its location in the abdomen of a human volunteer at different time intervals (hours). Note: The location of the ORS is represented with an arrow.

This clinical x-ray model is a good method to evaluate the gastric residence time of gastroretentive drug delivery systems. The representative images of the in vivo radiographic studies with the ORS are shown in Figure 6. The raft system is well retained in the stomach proximal to the absorption index and susceptible to releasing the drugs in a controlled, sustained manner. The x-ray images suggested that ORS can float for 8 h and that the optimized formula didn’t adhere to the gastric mucosa. The difference in the buoyancy time between the in vitro and in vivo studies contributed to the incorporation of the high-density barium sulfate agent (4.48 g/cm^3^). However, the barium sulfate-containing raft formula met the requirements based on the optimal floating lag time standards which are less than 3 minutes and a total floating time of more than 8 hours (Kim et al., 2016; Moganti & Shivakumar, 2021; Rahamathulla et al., 2021).

The location of the ORS might change continually as a function of time. The photomicrographs of the x-ray represent the retention of the prepared floating raft over eight hours in the upper part of the stomach. After then, the ORS was found in the lower part for digestion and elimination. Hence, ORS exhibits superiority in bioavailability and the ability to release ETO/FAM sustainably for maximum pain relief by ETO and the highest gastric protection by FAM.

### 4.4. In vivo pharmacokinetics in human volunteers

**Figure 7.**
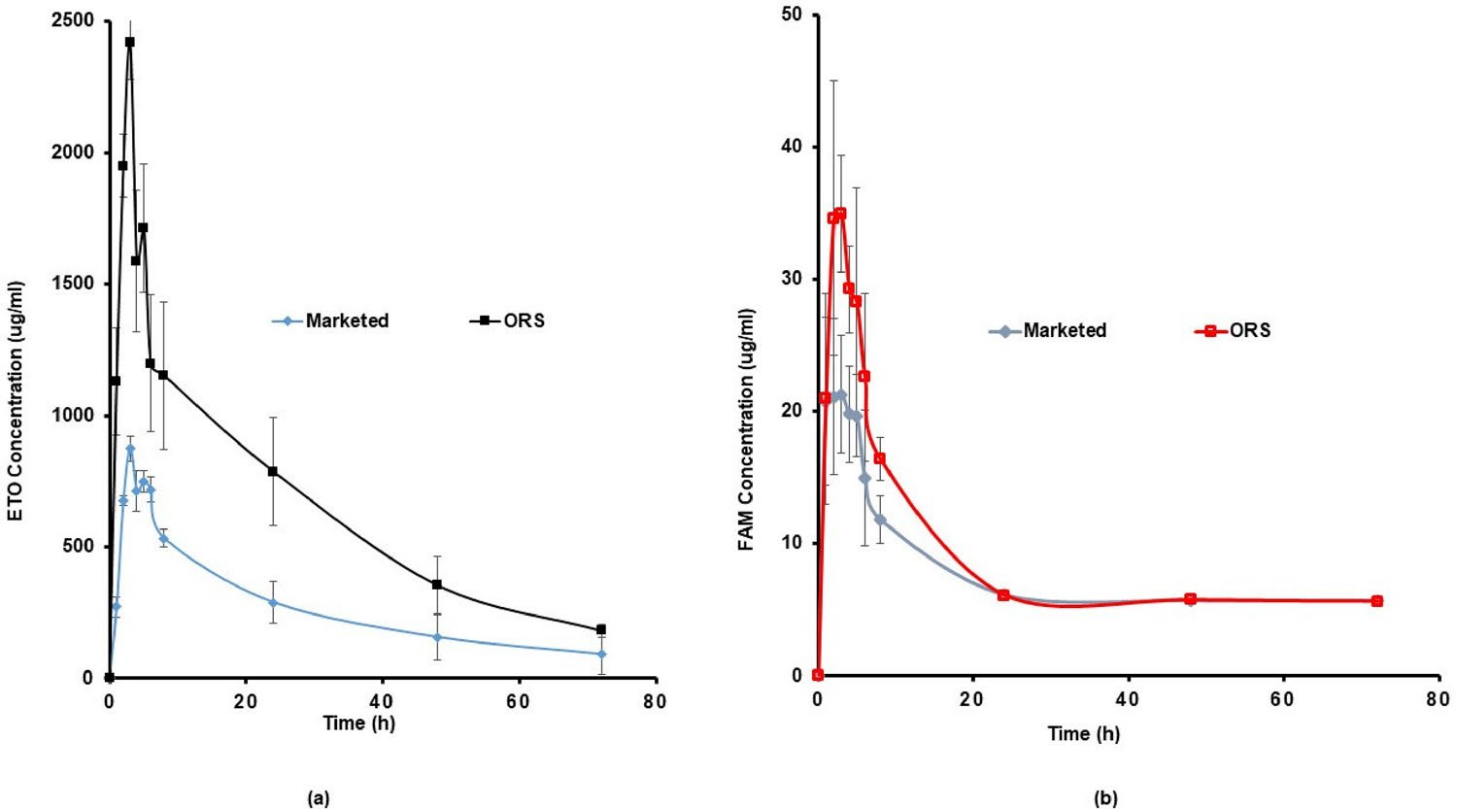
Average plasma concentration-time profiles after single oral administration of the ORS formulation, the marketed ETO (Arcoxia^®^), and the marketed FAM (Antodine^®^) to six human volunteers. Each point represents the mean values ± standard deviation (*n* = 6).

The results of plasma ETO/FAM concentrations at different time intervals, after administration of Arcoxia^®^, Antodine^®^, and ORS are illustrated in Figure 7. The different parameters were compared using one-way variance analysis (ANOVA). A value of *P* <0.05 was considered statistically significant. The pharmacokinetic parameters were derived from the plasma concentration versus the time profile of all the subjects (Rr et al., 2018), and the results are shown in Table 6. The average peak plasma concentration (C_max_) of ETO released from ORS was found to be 2418.578 ± 137.885 *μ*g.ml^−1^, which was higher than that of the marketed product Arcoxia^®^ (874.33 ± 18.01 *μ*g.ml^−1^) with an almost 2.7-fold increase in C_max_ of ETO. T_max_; the time required to reach the maximum plasma concentration of both systems was nearly similar (3 h for the marketed and ORS), this suggests that the absorption rate from both systems was identical (Rr et al., 2018). The AUC_0−∞_ of Arcoxia and ORS was found to be 23423.25 ± 2961.091 *μ*g.h/ml and 54249.444 ± 8767.933 *μ*g.h/ml, respectively. A statistically significant difference (*P* <0.05) among the different pharmacokinetic parameters of Arcoxia^®^ and ORS was observed. The AUC_0−∞_ was found to be 2.3 times increase in ORS than that of Arcoxia^®^.

**Table 6.** Pharmacokinetic parameters for Arcoxia^®^ (60 mg), Antodine^®^ (20 mg), and ORS (comprising 60 mg of ETO and 20 mg of FAM) after oral administration to six healthy volunteers (*n* = 6).

Parameter	Marketed		ORS	
	Arcoxia^®^	Antodine^®^	ETO	FAM
C_max_ (ng/ml)	874.33 ± 18.01	23.29 ± 6.50	2418.58 ± 137.89	39.08 ± 10.36
T_max_ (h)	3.00 ± 0.00	2.50 ± 1.38	3.00 ± 0.00	2.50 ± 0.55
AUC_(0-72)_ (ng.h/ml)	19859.26 ± 1572.69	558.92 ± 49.52	47401.54 ± 10043.83	658.41 ± 49.77
AUC_(0-∞)_ (ng.h/ml)	23423.25 ± 2961.09	1173.29 ± 88.39	54249.44 ± 8767.93	1067.24 ± 32.79
MRT (h)	22.95 ± 1.84	28.23 ± 1.96	22.55 ± 2.67	24.87 ± 1.58
K_el_ (1/h)	0.0273 ± 0.0003	0.0096 ± 0.0021	0.0295 ± 0.0071	0.0139 ± 0.0009
F%			238.69	117.80

Abbreviations: ORS, optimum raft system; T_max_, time to reach plasma concentration (C_max_); AUC, area under plasma concentration-time curve; MRT, mean residence time; K_el_, elimination rate constant.

These pharmacokinetic analyses of the plasma level data demonstrated that the oral bioavailability of ETO was enhanced upon oral administration of ORS than Arcoxia^®^ in the volunteer stomachs. The increase in the oral bioavailability might be attributed to the prolonged residence time of the delivery system, the solubility increase, the dissolution rate enhancement, and the higher absorption window of the ETO that improved its bioavailability. On the other hand, the average peak plasma concentration (C_max_) of FAM was 39.081 ± 10.357 *μ*g.ml^−1^ and is larger than the C_max_ of the market, that is 23.287 ± 6.499 *μ*g.ml^−1^. T_max_ of both the ORS and the marketed product was egalitarian (2.5 h for the marketed and ORS) (Bello, 2012), and as discussed before, this suggests that the rate of absorption from both systems was similar. These results served the main aim of the study to design the raft system and allow a superior release of FAM over ETO for gastric protection followed by a sustained release of both drugs for almost 8 h to achieve the maximum pain analgesia effect. When the AUC_0−∞_ of FAM from Antodine and ORS was compared, there was no significant difference between both values 1173.295 ± 88.388 *μ*g.h/ml and 1067.235 ± 32.787 *μ*g.h/ml.

## 5. Conclusion

ETO/FAM was prosperously formulated in the raft floating system using KGL as a novel polysaccharide fiber in the system. The optimized system possesses an optimum viscosity that allowed easy swallowing of the system as a liquid dosage form that then exhibits a rapid transition into a viscous floated gel with minimum FLT and GLT. The in vitro release studies allowed the superior release of the FAM to decrease stomach acidity and minimize the common side effect of ETO, followed by the release of ETO for pain analgesia. The pharmacokinetic results revealed that the optimum raft system not only was successful in a controllable release of both drugs; the analgesic and the antacid through the administration of a single dose unit for almost 8 h but also increased the bioavailability of etoricoxib as compared to the marketed formulation and lessened its GIT side effect. Thus, a promising raft-forming system has been achieved that would perfectly fit special populations such as geriatrics and pediatrics who struggle with swallowing the solid dosage forms of analgesic drugs, thus improving efficacy due to enhanced patient compliance.

## References

[CIT0001] Aanisah N, Wardhana YW, Chaerunisaa AY, Budiman A. (2022). Review on modification of glucomannan as an excipient in solid dosage forms. Polymers 14:1. 10.3390/polym14132550 PMC9269564 35808596

[CIT0002] Aashigari S, G, R, S, S, Vykuntam U, Potnuri N. (2019). Stability studies of pharmaceutical products. World J Pharm Res 8:479–16.

[CIT0003] Abou Youssef NAH, Kassem AA, El-Massik MAE, Boraie NA. (2015). Development of gastroretentive metronidazole floating raft system for targeting Helicobacter pylori. Int J Pharm 486:297–305. 25843757 10.1016/j.ijpharm.2015.04.004

[CIT0004] Abouelatta SM, Aboelwafa AA, El-Gazayerly ON. (2018). Gastroretentive raft liquid delivery system as a new approach to release extension for carrier-mediated drug. Drug Delivery 25:1161–74. 29792353 10.1080/10717544.2018.1474969 PMC6058684

[CIT0049] Abdelmonem R, Elhabal SF, Abdelmalak NS, et al. (2021). Formulation and characterization of acetazolamide/carvedilol niosomal gel for glaucoma treatment. In Vitro and in Vivo Study. Pharmaceutics 13(2):221–41. 33562785 10.3390/pharmaceutics13020221 PMC7915822

[CIT0005] Ahmadi M, Bekeschus S, Weltmann K-D, et al. (2022). Non-steroidal anti-inflammatory drugs: recent advances in the use of synthetic COX-2 inhibitors. RSC Med. Chem 13:471–96. 35685617 10.1039/d1md00280e PMC9132194

[CIT0006] Ain S, Kumar B, Pathak K. (2017). Development and characterization of controlled release famotidine matrix tablets containing complexes. Int J App Pharm 9:38–46.

[CIT0007] Alvarez-Manceñido F, Landin M, Lacik I, Martínez-Pacheco R. (2008). Konjac glucomannan and konjac glucomannan/xanthan gum mixtures as excipients for controlled drug delivery systems. Diffusion of small drugs. Int J Pharm 349:11–8. 17804182 10.1016/j.ijpharm.2007.07.015

[CIT0008] Arfan AR, Ilmiawati A, Sugita P. (2022). Optimization and synthesis of etoricoxib-loaded low molecular weight chitosan nanoparticles. Ciência Rural 52.

[CIT0009] Behera SS, Ray RC. (2016). Konjac glucomannan, a promising polysaccharide of Amorphophallus konjac K. Int J Biol Macromol 92:942–56. 27481345 10.1016/j.ijbiomac.2016.07.098

[CIT0010] Bello AE. (2012). DUEXIS(®) (ibuprofen 800 mg, famotidine 26.6 mg): A new approach to gastroprotection for patients with chronic pain and inflammation who require treatment with a nonsteroidal anti-inflammatory drug. Ther Adv Musculo 4:327–39. 10.1177/1759720X12444710 PMC3458616 23024710

[CIT0011] Bello AE, Kent JD, Holt RJ. (2015). Gastroprotective efficacy and safety of single-tablet ibuprofen/famotidine vs ibuprofen in older persons. Phys Sportsmed 43:193–9. 26165391 10.1080/00913847.2015.1066229

[CIT0012] Bhavsar DN. (2012). Advances in GRDDS: raft forming system a review. J. Drug Delivery Ther 2.

[CIT0013] Birk JW, Myers M. (2009). A fixed dose combination of ibuprofen and famotidine. Expert Opin Invest Drugs 18:1385–91. 10.1517/13543780903160641 19659445

[CIT0014] Brenner T, Tuvikene R, Fang Y, et al. (2015). Rheology of highly elastic iota-carrageenan/kappa-carrageenan/xanthan/konjac glucomannan gels. Food Hydrocoll 44:136–44.

[CIT0015] Das A, Nayak AK, Mohanty B, Panda S. (2011). Solubility and dissolution enhancement of etoricoxib by solid dispersion technique using sugar carriers. ISRN Pharm 2011:819765. 22389861 10.5402/2011/819765 PMC3263729

[CIT0016] Dash S, Murthy PN, Nath L, Chowdhury P. (2010). Kinetic modeling on drug release from controlled drug delivery systems. Acta Poloniae Pharma 67:217–23. 20524422

[CIT0017] Deeks ED. (2013). Fixed-dose ibuprofen/famotidine: a review of its use to reduce the risk of gastric and duodenal ulcers in patients requiring NSAID therapy. Clin Drug Investig 33:689–97. 10.1007/s40261-013-0113-x 23881568

[CIT0018] Devaraj RD, Reddy CK, Xu B. (2019). Health-promoting effects of konjac glucomannan and its practical applications: a critical review. Int J Biol Macromol 126:273–81. 30586587 10.1016/j.ijbiomac.2018.12.203

[CIT0019] Devi R, Agarwal S. (2019). Some multifunctional lipid excipients and their pharmaceutical applications. Int J Pharm Pharm Sci 11:1–7.

[CIT0020] Dranca F, Oroian M. (2019). Optimization of pectin enzymatic extraction from Malus domestica “Fălticeni” Apple Pomace with Celluclast 1.5L. Molecules (Basel, Switzerland) 24(11):2158–71. 31181702 10.3390/molecules24112158 PMC6600438

[CIT0021] El Nabarawi MA, Teaima MH, El-Monem A, et al. (2017). Formulation, release characteristics, and bioavailability study of gastroretentive floating matrix tablet and floating raft system of Mebeverine HCl. Drug Des Devel Ther 11:1081–93. 10.2147/DDDT.S131936 PMC5388234 28435220

[CIT0022] ELhabal SF, Elwy HM, Hassanin S, et al. (2022). Biosynthesis and characterization of gold and copper nanoparticles from Salvadora persica fruit extracts and their biological properties. Int J Nanomed 17:6095–112. 10.2147/IJN.S385543 PMC9741820 36514376

[CIT0023] Farag M, Bahra A. (2022). Etoricoxib and celecoxib sensitive indomethacin-responsive headache disorders. Headache 62:383–8. 35277974 10.1111/head.14282

[CIT0024] Farrow C, Heidel J, Maloney J, Rogers J. (2004). Scalar equations for synchronous Boolean networks with biological applications. IEEE Trans Neural Netw 15:348–54. 15384528 10.1109/TNN.2004.824262

[CIT0025] Gawkowska D, Cybulska J, Zdunek A. (2018). Structure-related gelling of pectins and linking with other natural compounds: a review. Polymers 10:762. 30960687 10.3390/polym10070762 PMC6404037

[CIT0026] Hamdani J, Moës AJ, Amighi K. (2002). Development and evaluation of prolonged release pellets obtained by the melt pelletization process. Int J Pharm 245:167–77. 12270253 10.1016/s0378-5173(02)00348-4

[CIT0027] Ibrahim HK. (2009). A novel liquid effervescent floating delivery system for sustained drug delivery. Drug Discov Ther 3:168–75. 22495603

[CIT0028] Islam MS, Narurkar MM. (2011). Solubility, stability and ionization behavior of famotidine. J Pharm Pharmacology 45:682–6. 10.1111/j.2042-7158.1993.tb07088.x 7901363

[CIT0029] Jaroenkietkajorn U, Mahapokai S, Wichchukit S. (2018). Effect of pectin and gellan gum on particles and added solids’ stability in drinking fermented goat milk. 1–6.

[CIT0030] Ju Z, Li M, Xu J, et al. (2022). Recent development on COX-2 inhibitors as promising anti-inflammatory agents: the past 10 years. Acta Pharm Sin B 12:2790–807. 35755295 10.1016/j.apsb.2022.01.002 PMC9214066

[CIT0031] Kim JS, Cha KH, Kang SY, et al. (2016). In vivo gastric residence and gastroprotective effect of floating gastroretentive tablet of DA-9601, an extract of Artemisia asiatica, in beagle dogs. Drug Des Devel Ther 10:1917–25. 10.2147/DDDT.S102918 PMC4907637 27354765

[CIT0032] Kirschneck C, Küchler EC, Wolf M, et al. (2019). Effects of the highly COX-2-selective analgesic NSAID etoricoxib on human periodontal ligament fibroblasts during compressive orthodontic mechanical strain. Mediat Inflamm 2019:1–14. 10.1155/2019/2514956 PMC6431464 30983880

[CIT0033] la Torre LF, Franco-González DL, Brennan-Bourdon LM, et al. (2021). Analgesic efficacy of etoricoxib following third molar surgery: a meta-analysis. Behav Neurol 2021:9536054. 34539935 10.1155/2021/9536054 PMC8445708

[CIT0034] Leiman DA, Riff BP, Morgan S, et al. (2017). Alginate therapy is effective treatment for GERD symptoms: a systematic review and meta-analysis. Dis Esophagus 30:1–9. 10.1093/dote/dow020 PMC6036656 28375448

[CIT0035] Lewis GA, Mathieu D, Phan-Tan-Luu R. (1998). Pharma Experimental Des. Boca Raton: CRC Press.

[CIT0036] Li B, Xia J, Wang Y, Xie B. (2005). Grain-size effect on the structure and antiobesity activity of konjac flour. J Agric Food Chem 53:7404–7. 16159165 10.1021/jf050751q

[CIT0037] Liu Z-L, Hu H, Zhuo R-X. (2004). Konjac glucomannan-graft-acrylic acid hydrogels containing azo crosslinker for colon-specific delivery. J Polym Sci A, Polym Chem 42:4370–8.

[CIT0038] Martina SD, Vesta KS, Ripley TL. (2005). Etoricoxib: a highly selective COX-2 inhibitor. Ann Pharmacother 39:854–62. 15827069 10.1345/aph.1E543

[CIT0039] Menache A. (2000). Healthy human volunteers and informed consent. Med Law 19:532–5. https://pubmed.ncbi.nlm.nih.gov/11143887/11143887

[CIT0040] Mishra R, Datt M, Pal K, Banthia A. (2008). Preparation and characterization of amidated pectin based hydrogels for drug delivery system. J Mater Sci – Mater Med 19:2275–80. 18058200 10.1007/s10856-007-3310-4

[CIT0041] Moganti M, Shivakumar HN. (2021). Oral raft forming in situ gelling system for site specific delivery of calcium. J Drug Delivery Sci Technol 61:102113.

[CIT0042] Morris E, Nishinari K, Rinaudo M. (2012). Gelation of gellan – a review. Food Hydrocoll 28:373–411.

[CIT0043] Mostafavi A, Emami J, Varshosaz J, et al. (2011). Development of a prolonged-release gastroretentive tablet formulation of ciprofloxacin hydrochloride: pharmacokinetic characterization in healthy human volunteers. Int J Pharm 409:128–36. 21371548 10.1016/j.ijpharm.2011.02.035

[CIT0044] Mummaneni V, Vasavada RC. (1990). Solubilization and dissolution of famotidine from solid glass dispersions of xylitol. Int J Pharm 66:71–7.

[CIT0045] Muthukumar M, Raju DM, Rajendran M. (2003). Optimization of mix proportions of mineral aggregates using Box Behnken design of experiments. Cem Concr Compos 25:751–8.

[CIT0050] Pereira R, Carvalho A, Vaz DC, et al. (2013). Development of novel alginate based hydrogel films for wound healing applications. Int J Biol Macromol 52:221–30. 23059189 10.1016/j.ijbiomac.2012.09.031

[CIT0047] Phillips, G. O., Williams, P. A. (Eds.). (2009). Handbook of Hydrocolloids. 2nd ed. Abington Hall, Granta Park: Woodhead Publishing.

[CIT0048] Prajapati VD, Jani GK, Khutliwala TA, Zala BS. (2013). Raft forming system-an upcoming approach of gastroretentive drug delivery system. Journal of Controlled Release: Official Journal of the Controlled Release Society 168:151–65. 23500062 10.1016/j.jconrel.2013.02.028

[CIT0051] Rahamathulla M, Saisivam S, Alshetaili A, et al. (2021). Design and evaluation of losartan potassium effervescent floating matrix tablets: In vivo x-ray imaging and pharmacokinetic studies in Albino Rabbits. Polymers 13:3476–92. 34685235 10.3390/polym13203476 PMC8538939

[CIT0052] Rajinikanth PS, Balasubramaniam J, Mishra B. (2007). Development and evaluation of a novel floating in situ gelling system of amoxicillin for eradication of Helicobacter pylori. Int J Pharm 335:114–22. 17141986 10.1016/j.ijpharm.2006.11.008

[CIT0053] Rosiaux Y, Jannin V, Hughes S, Marchaud D. (2014). Solid lipid excipients—matrix agents for sustained drug delivery. JCR 188:18–30. 10.1016/j.jconrel.2014.06.004 24929038

[CIT0055] Saha D, Bhattacharya S. (2010). Hydrocolloids as thickening and gelling agents in food: a critical review. J Food Sci Technol 47:587. 23572691 10.1007/s13197-010-0162-6 PMC3551143

[CIT0057] Sapkal SB, Adhao VS, Thenge RR, et al. (2020). Formulation and Characterization of Solid Dispersions of Etoricoxib Using Natural Polymers. Solid dispersions of etoricoxib using natural polymers. Turkish J Pharma Sci 17:7–19. 10.4274/tjps.galenos.2018.04880 PMC7227871 32454755

[CIT0058] Shah KU, Khan GM. (2012). Regulating drug release behavior and kinetics from matrix tablets based on fine particle-sized ethyl cellulose ether derivatives: an *in vitro* and *in vivo* evaluation. Sci World J 2012:842348–56. 10.1100/2012/842348 PMC3350987 22649325

[CIT0059] Shah JC, Sadhale Y, Chilukuri DM. (2001). Cubic phase gels as drug delivery systems. Adv Drug Delivery Rev 47:229–50. 10.1016/s0169-409x(01)00108-9 11311994

[CIT0060] Srinivas L, Sagar S. (2022). Design, optimization, and evaluation of raft forming gastro retentive drug delivery system of lafutidine using Box–Behnken design. Int J Appl Pharma 14:266–74.

[CIT0061] Sugano K. (2013). Single-tablet double-dose famotidine plus ibuprofen decreases endoscopic upper GI ulcers compared with ibuprofen alone. EBM 18:26–7. 22773762 10.1136/ebmed-2012-100711

[CIT0062] Szekalska M, Puciłowska A, Szymańska E, et al. (2016). Alginate: current use and future perspectives in pharmaceutical and biomedical applications. Int J Poly Sci 2016:2016–33.

[CIT0063] Taha AS. (2015). The ibuprofen-famotidine combined pill—a promise fulfilled. Curr Med Res Opin 31:421–2. 25713982 10.1185/03007995.2015.1010037

[CIT0064] Teaima MH, Abdel Hamid MM, Shoman NA, et al. (2020). Formulation, characterization and comparative pharmacokinetic study of bupropion floating raft system as a promising approach for treating depression. J Pharm Sci 109:3451–61. 32835701 10.1016/j.xphs.2020.08.011

[CIT0065] Teaima M, Hamid MMA, Shoman NA, et al. (2020). Promising swellable floating bupropion tablets: formulation, in vitro/in vivo evaluation and comparative pharmacokinetic study in human volunteers. Drug Des Devel Ther 14:2741. 10.2147/DDDT.S258571 PMC7368561 32764875

[CIT0054] Tjandrawinata RR, Setiawati A, Nofiarny D, et al. (2018). Pharmacokinetic equivalence study of nonsteroidal anti-inflammatory drug etoricoxib. Clin Pharmacology Adv Appl 10:43–51. 10.2147/CPAA.S161024 PMC5896653 29670410

[CIT0066] Vaughn CJ. (2012). Drugs and lactation database: LactMed. J Elect Res Med Lib 9:272–7.

[CIT0067] Wang S-Q, Huang G-Q, Du Y-L, Xiao J-X. (2017). Modification of konjac glucomannan by reduced-pressure radio-frequency air plasma. Int J Food Eng 13:20160377.

[CIT0068] Wang L-X, Lee A-R, Yuan Y, et al. (2020). Preparation and FTIR, Raman and SEM characterizations of konjac glucomannan-KCl electrogels. Food Chem 331:127289. 32569966 10.1016/j.foodchem.2020.127289

[CIT0069] Wei X, Cui C, Fan C, et al. (2022). Injectable hydrogel based on dodecyl-modified N-carboxyethyl chitosan/oxidized konjac glucomannan effectively prevents bleeding and postoperative adhesions after partial hepatectomy. Int J Biol Macromol 199:401–12. 34999041 10.1016/j.ijbiomac.2021.12.193

[CIT0070] WMA – The World Medical Association – WMA Declaration of Helsinki – Ethical Principles for Medical Research Involving Human Subjects. (n.d.). Available at: https://www.wma.net/policies-post/wma-declaration-of-helsinki-ethical-principles-for-medical-research-involving-human-subjects/10.1191/0969733002ne486xx 16010903

[CIT0071] Xu Z, Zhang L, Bentil SA, Bratlie KM. (2021). Gellan gum-gelatin viscoelastic hydrogels as scaffolds to promote fibroblast differentiation. Mater Sci Eng C Mater Biol Appl 129:112370. 34579889 10.1016/j.msec.2021.112370

[CIT0072] Yamamoto A, Itoh T, Nasu R, Nishida R. (2014). Sodium alginate ameliorates indomethacin-induced gastrointestinal mucosal injury via inhibiting translocation in rats. WJG 20:2641–52. 24627600 10.3748/wjg.v20.i10.2641 PMC3949273

[CIT0073] Yu H, Huang A, Xiao C. (2006). Characteristics of konjac glucomannan and poly(acrylic acid) blend films for controlled drug release. J Appl Polym Sci 100:1561–70.

[CIT0074] Yuan Y, Hunt RH. (2007). Global gastrointestinal safety profile of etoricoxib and lumiracoxib. Curr Pharm Des 13:2237–47. 17691997 10.2174/138161207781368792

[CIT0075] Zhang Y, Huo M, Zhou J, et al. (2010). DDSolver: an add-in program for modeling and comparison of drug dissolution profiles. AAPS J 12:263–71. 20373062 10.1208/s12248-010-9185-1 PMC2895453

[CIT0076] Zhang Y, Huo M, Zhou J, Xie S. (2010). PKSolver: an add-in program for pharmacokinetic and pharmacodynamic data analysis in Microsoft Excel. Comput Meth Prog Biomed 99:306–14. 10.1016/j.cmpb.2010.01.007 20176408

[CIT0077] Zhang W, Ren X, Zhang L, Chen J. (2022). Preparation and performance of thickened liquids for patients with konjac glucomannan-mediated dysphagia. Molecules (Basel, Switzerland) 27:2194–213. 35408593 10.3390/molecules27072194 PMC9000327

[CIT0078] Zhao Y, Jayachandran M, Xu B. (2020). In vivo antioxidant and anti-inflammatory effects of soluble dietary fiber konjac glucomannan in type-2 diabetic rats. Int J Biol Macromol 159:1186–96. 32428590 10.1016/j.ijbiomac.2020.05.105

[CIT0079] Zhao C-X, Wang J-W, Gong M. (2020). Efficacy and safety of alginate formulations in patients with gastroesophageal reflux disease: a systematic review and meta-analysis of randomized controlled trials. Eur Rev Med Pharmacology Sci 24:11845–57. 10.26355/eurrev_202011_23841 33275256

